# The osteosarcoma immune microenvironment in progression: PLEK as a prognostic biomarker and therapeutic target

**DOI:** 10.3389/fimmu.2025.1651858

**Published:** 2025-08-15

**Authors:** Yunpeng Zou, Jianning Kang, Shaopeng Zhu, Xuechen Ren, Zheng Li, Jiayao Niu, Xuanzhe Qin, Hongbo Li, Lu Xiang, Wei Jiang, Jiangbo Zhong, Ying Zhang, Kai Zhao

**Affiliations:** ^1^ School of Clinical Medicine, Shandong Second Medical University, Weifang, Shandong, China; ^2^ Central Hospital Affiliated to Shandong First Medical University, Shandong First Medical University & Shandong Academy of Medical Sciences, Jinan, Shandong, China; ^3^ Jinan Central Hospital, Shandong University, Jinan, Shandong, China; ^4^ The Second Clinical Medical School, Lanzhou University, Lanzhou, China; ^5^ The Second Affiliated Hospital of Shandong University of Traditional Chinese Medicine, Jinan, China

**Keywords:** osteosarcoma, immune microenvironment, molecular biomarkers, multi-omics analysis, single-cell RNA sequencing, PLEK

## Abstract

**Introduction:**

Osteosarcoma (OS) is a malignant bone tumor with high metastatic potential and poor long-term survival. The tumor immune microenvironment and metabolic reprogramming are increasingly recognized as key drivers of OS progression, yet the molecular links between these systems remain unclear. This study aimed to identify immune-metabolic biomarkers in OS, focusing on pleckstrin (PLEK) as a potential regulatory hub.

**Methods:**

We conducted differential expression and survival analyses using OS transcriptomic datasets and TCGA/GTEx data. Protein–protein interaction networks, GO/KEGG enrichment, and CytoHubba algorithms identified core hub genes. Tumor-infiltrating immune cells were profiled via TIMER. Single-cell RNA-seq (GSE162454) was used for immune and metabolic landscape mapping. PLEK was further validated by qRT-PCR and Western blot in OS samples, and its function assessed via siRNA knockdown in macrophages within TME co-cultured with OS cells. Cell proliferation, migration, and invasion assays evaluated phenotypic effects in OS cells.

**Results:**

Nine hub genes were identified, with PLEK significantly upregulated in OS tissues. High PLEK expression correlated with improved survival and increased infiltration of macrophages, dendritic cells, and CD4^+^ T cells. Single-cell analysis showed PLEK enrichment in macrophage-dominated clusters with active glycolytic and oxidative phosphorylation pathways. Downregulation of PLEK in macrophages enhanced OS cell proliferation, migration and invasion. These findings suggest PLEK is linked to a pro-immune, metabolically active microenvironment and may act as a tumor suppressor.

**Discussion:**

Our study identifies PLEK as a prognostic biomarker and functional regulator in OS. It promotes an immune-infiltrated, metabolically active tumor microenvironment and is associated with attenuated malignant phenotypes *in vitro*. These findings highlight PLEK as a promising target for immunometabolic modulation in OS.

## 1 Introduction

OS, a highly malignant bone tumor derived from mesenchymal cells, is the most common primary bone malignancy in adolescents as well as in elderly patients with bone deformities such as Paget’s disease (1). OS occurs predominantly in the epiphyses of the long bones, with high heterogeneity and invasiveness, and is highly susceptible to metastasis (2). Currently, the standard treatment regimen is presurgical chemotherapy-surgery-adjuvant postsurgical chemotherapy (including high-dose methotrexate, doxorubicin and cisplatin), which significantly improves five-year survival in patients with non-metastatic OS (3). Nevertheless, for patients afflicted with advanced and metastatic OS, the overall survival rate persists below 30% even with adjuvant chemotherapy (4, 5). Unfortunately, the mechanisms underlying the pathogenesis and progression of OS, including chemoresistance, susceptibility to relapse, and distant metastasis, are still unclear.

Early diagnosis plays a critical role in the prognosis of OS, but current diagnostic methods, including imaging techniques and serum biochemical markers, have limitations of insufficient specificity and sensitivity (6). Therefore, it is imperative to elucidate the mechanisms underlying the pathogenesis and progression of OS, identify molecular biomarkers and promote precision medicine for OS (7). New advances in genome-wide association studies (GWAS) have led to the identification of multiple cancer susceptibility genes associated with OS pathogenesis, progression and prognosis, providing new insights into the molecular pathology of OS (8).

A mounting body of research has demonstrated that the tumor microenvironment (TME) plays a significant role in influencing OS progression, immune evasion, and chemoresistance (9). The TME is a highly complex system containing tumor cells and a large number of non-tumor cells, which contribute to OS invasion and metastasis (10). The TME of OS is markedly immunosuppressive, as evidenced by the dysfunction of anti-tumor immune cells. Moreover, immunosuppressive cells embedded in TME, like regulatory T cells (Tregs) and myeloid-derived suppressor cells (MDSCs), also exacerbates immune evasion, contributing to the lack of efficacy of immunotherapy (11).

In addition, OS development is accompanied by significant metabolic reprogramming to adapt to harsh conditions. Studies have shown that the Warburg effect in OS makes tumor cells dependent on glycolysis for energy generation even in an aerobic environment (12, 13). This metabolic shift fosters the rapid proliferation of tumor cells, and alters the immune environment and suppresses tumor-infiltrating immune cells (14). Furthermore, changes in lipid metabolism, amino acid metabolism, and mitochondrial function are also closely related to the progression of OS, which provides a novel direction for finding potential therapeutic targets (15, 16).

This study conducted bioinformatics analysis on multiple GEO and TCGA datasets, including functional enrichment analysis, protein interaction network construction, survival analysis, tumor-infiltrating immune cell (TIIC) assessment, and functional validation, to identify genes associated with the prognosis of OS and screen for potential prognostic biomarkers and therapeutic targets PLEK. Subsequently, a series of functional validation studies were conducted on this gene demonstrated that down-regulation of PLEK in macrophages enhanced the proliferation, migration and invasion of OS cells, providing a theoretical foundation for precision medicine in OS.

In conclusion, recent advances on the immune microenvironment and metabolic reprogramming in OS provide guidance for advancement of innovative therapeutic strategies. And it is expected that combining conventional chemotherapy with immune checkpoint inhibitors or drugs targeting metabolic pathways could enhance the therapeutic effect and improve patient prognosis. With a more profound comprehension of the intricate interactions among tumor cells, immune microenvironment and metabolic networks, the precision treatment of OS is becoming possible.

## 2 Methods

### 2.1 Collection and analysis of transcriptomic data

The Gene Expression Omnibus (GEO) is a functional genomics data repository, maintained by the National Center for Biotechnology Information (NCBI). To identify DEGs associated with OS, we selected three datasets: GSE12865 (17), GSE14359 (18), and GSE36001 (19). The inclusion criteria for the selected datasets were as follows: i) Tumor samples included primary or metastatic OS tissue. ii) Control samples included normal human bone tissue or osteoblasts. iii) Statistical thresholds for DEGs were p < 0.05 and |log2 fold change| > 1 iv) At least 10 overlapping DEGs in multiple datasets were required.

### 2.2 Identification of DEGs

DEGs were identified by GEO2R, an interactive web tool that compares two or more sample sets in GEO and ranks genes based on statistical significance. We used |log2FC| ≥ 1 and p < 0.05 as thresholds. Venn plots were generated using the Venn tool (http://bioinformatics.psb.ugent.be/webtools/Venn/) to visualize overlapping DEGs.

### 2.3 KEGG and GO pathway enrichment analysis

Gene Ontology (GO) was analyzed to explore the enrichment of DEGs in three major categories: biological process (BP), cellular component (CC) and molecular function (MF). Kyoto Encyclopedia of Genes and Genomes (KEGG) pathway analysis was used to investigate functional interactions at the molecular level (20). Both analyses were performed by the Database for Annotation, Visualization and Integrated Discovery (DAVID, http://david.ncifcrf.gov/), a widely used bioinformatics tool for systematic analysis of large-scale gene and protein datasets.

### 2.4 Analysis of protein-protein interaction network

A PPI network was created using the STRING (Search Tool for Retrieving Gene/Protein Interactions) database and visualized through Cytoscape software. We then identified the most critical hub genes using the Molecular Complexity Detection (MCODE), an algorithm that allows for highly interrelated genes for scoring and screening. Moreover, the CytoHubba algorithm was used to further identify hub genes based on different topological properties, which can detect key hub genes in the network, thus providing a comprehensive view of molecular interactions.

### 2.5 Gene expression analysis

Box plots were created from RNA sequencing (RNA-seq) data from The Cancer Genome Atlas (TCGA) and Genotype-Tissue Expression (GTEx) databases using the Gene Expression Profiling Interactive Analysis 2.0 (GEPIA2) platform (http://gepia2.cancer-pku.cn/). Gene expression values were normalized to transcripts per million (TPM), log2 transformed, and compared between tumor and normal tissues. Statistical significance was assessed via the Wilcoxon rank sum test, with significant differences indicated by an asterisk.

### 2.6 Prognostic analysis of DEGs

The prognostic significance of central genes was assessed using the Kaplan-Meier (KM) plotter (https://kmplot.com/analysis/), which integrates survival data from GEO and TCGA. OS patient samples were divided into high and low expression groups according to the expression levels of hub genes. Kaplan-Meyer survival curves, hazard ratios (HR), 95% confidence intervals (CI), and log-rank P-values were calculated to assess the potential prognostic value of centrality.

### 2.7 Tumor-infiltrating immune cell analysis

The abundance of TIIC in OS samples was inferred from the TIMER (https://cistrome.shinyapps.io/timer/) database, an integrated platform for immune cell infiltration analysis. TIMER used an inverse plethysmography approach to infer tumor-infiltrating immune cells the gene expression profiles of samples from various cancer types in the TCGA (B cells, CD8+ T cells, CD4+ T cells, macrophages, neutrophils and dendritic cells) abundance.

### 2.8 Single-cell RNA sequencing

Single cell suspensions were processed using the 10× Chromium Single Cell Platform (10× Genomics, 30 v3 chemistry). Individual cell mRNAs were captured, cDNAs synthesized, and libraries constructed according to the manufacturer’s protocol. Sequencing was performed on the NovaSeq 6000 platform (Illumina) with 150 bp paired-end reads. Data were normalized using logarithmic transformation. Raw data were processed using the Cell Ranger 3.0.1 pipeline for alignment, data processing, and initial clustering. Single-cell data were obtained from GEO (GSE162454). scMetabolism (v0.2.1) pipeline was used to assess the metabolic diversity of each cluster. It scores each cluster and calculates activity scores for metabolic pathways based on single-cell matrix files.

### 2.9 RNA extraction and quantitative real-time PCR

Total RNA was extracted using TRIzol reagent (Invitrogen, CA, USA), and then reverse-transcribed into cDNA using the Evo M-MLV RT Premix Kit (Accurate Biology, Hunan, China). Real-time quantitative reverse transcription polymerase chain reaction (qRT-PCR) was performed using the LightCycler^®^480 (Roche, USA) system and SYBR^®^Green Premix Pro Taq HS qPCR Kit (Accurate Biology, Hunan, China). The procedures above were performed according to the manufacturer’s instructions.

### 2.10 Western blot analysis

Protein samples were prepared from the collected tissues, subjected to SDS-polyacrylamide gel electrophoresis, and transferred to nitrocellulose membranes. The membranes were blocked with 5% non-fat milk at room temperature for 2 hours, followed by incubation with the purchased primary antibody overnight. Subsequently, the membranes were washed three times with Tris-buffered saline (TBS) containing Tween 20, and then incubated with the secondary antibody at room temperature for 1–2 hours. After washing the membrane three times, the protein expression levels were detected using chemiluminescence reagents. The antibodies used were as follows: anti-CYBB antibody (1:1000; ABclonal, A19701); anti-PLEK antibody (1:1000; ABclonal, A6305); anti-Vinculin (1:10000; Abways, CY5164).

### 2.11 Cell counting kit-8 assays

Cell proliferation was evaluated using CCK-8 assays (Yeasen Biotechnology). Briefly, OS cells (1×10^5^/well) were plated in 96-well plates and cultured for 24–96 h. After adding 10 μL CCK-8 solution per well, plates were incubated at 37°C for 1 h. Absorbance at 450 nm was measured using a microplate reader (SpectraMax i3x).

### 2.12 Colony formation

Cells were seeded at a density of 600 cells/well in 6-well plates and cultured for 10–14 days, with medium changes performed as needed. Following colony formation, cells were washed twice with PBS, fixed with 4% paraformaldehyde (15min), and stained with 1% crystal violet (30min). Colonies containing >50 cells were counted under a light microscope. All experiments were conducted in triplicate.

### 2.13 EdU assays

Cell proliferation was analyzed using the Cell Light™ EdU Apollo567 *In Vitro* Kit (RiboBio). Briefly, OS cells were co-cultured seeded in 96-well plates for 48 h, followed by incubation with 50 μM EdU for 2 h at 37°C. Cells were then fixed with 4% formaldehyde (30 min), treated with 2 mg/mL glycine (5min), and permeabilized with 0.5% Triton X-100 (10min). After PBS washing, samples were incubated with Apollo reaction cocktail (30min), washed twice with 0.5% Triton X-100, and counterstained with Hoechst 33342 (30min). Fluorescence images were acquired using an Olympus microscope.

### 2.14 Transwell evaluation

Cell migration and invasion were assessed using Transwell chambers (8 µm pore size; Corning). For invasion assays, chambers were pre-coated with 50 µL of 1:8 diluted Matrigel (Corning), whereas uncoated chambers were used for migration assays. Cells (1×10^5^) suspended in 200 µL serum-free medium were seeded in the upper chamber, while the lower chamber contained medium supplemented with 10% FBS as a chemoattractant. After 24 h incubation at 37°C, cells that migrated/invaded to the lower chamber were fixed with 4% paraformaldehyde, stained with 0.1% crystal violet (1h, RT), and quantified by counting five random fields per well under a microscope. All experiments were performed in triplicate.

## 3 Results

### 3.1 Identification of DEGs

**Figure 1:**
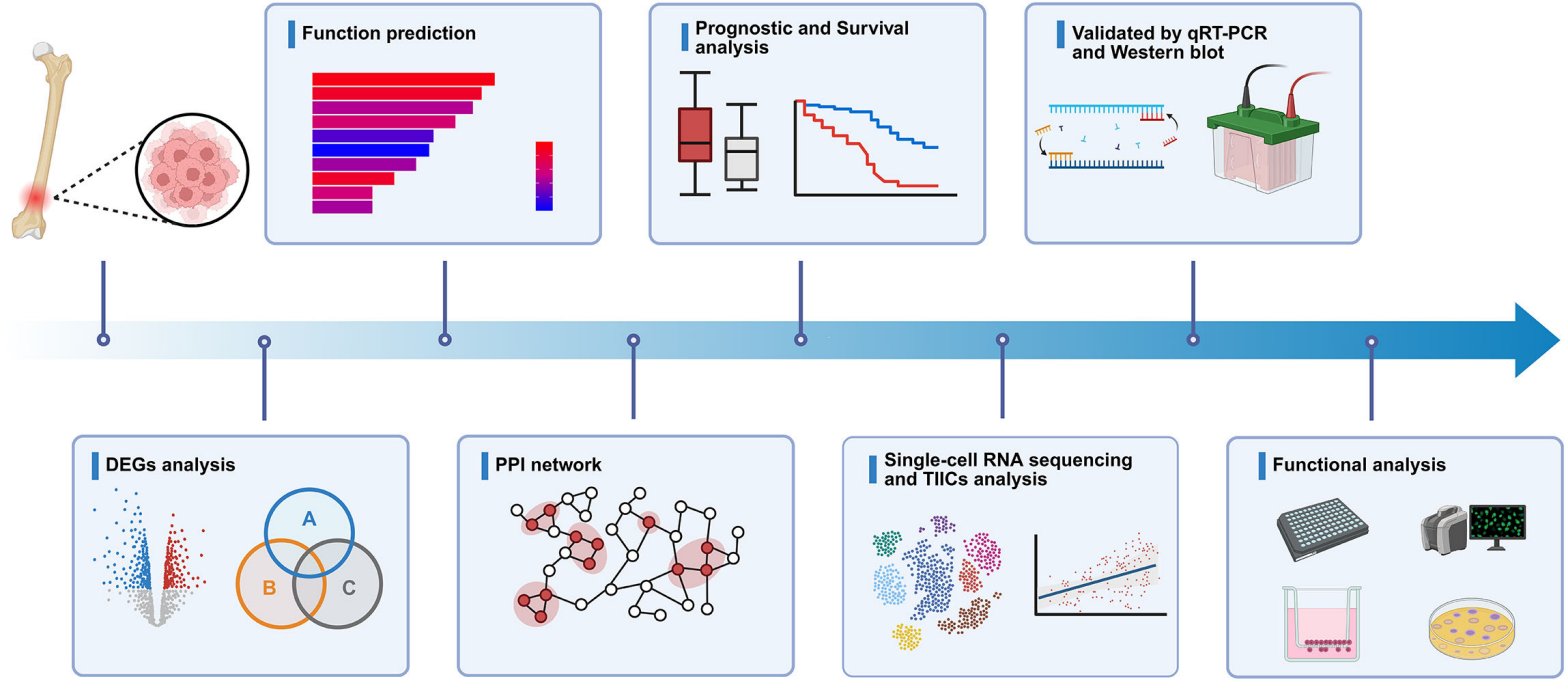
Schematic diagram of the experiment. Schematic representation of the study workflow, including the systematic identification and subsequent validation of hub genes.

**Figure 2:**
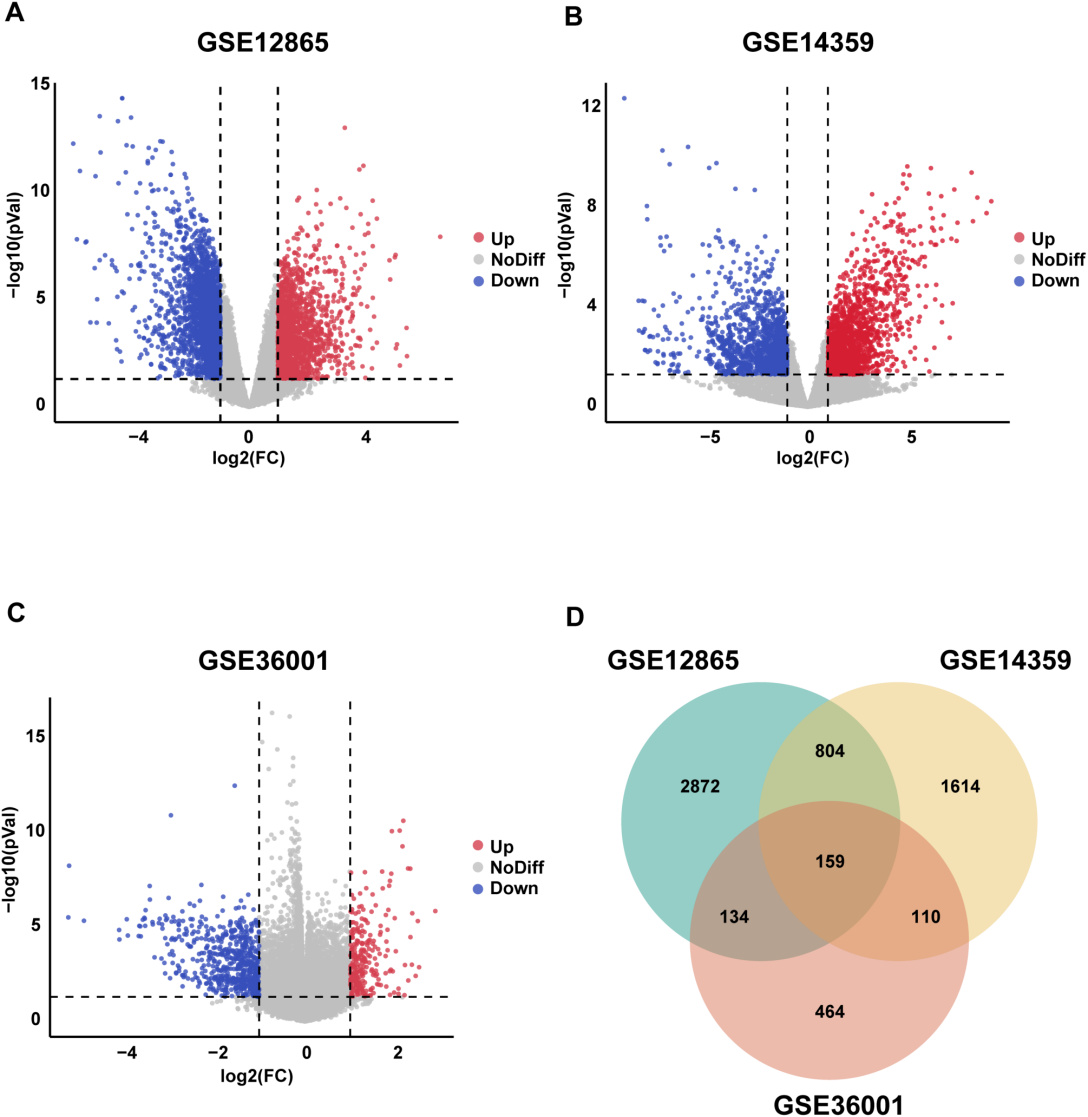
Identification of DEGs in OS. **(A–C)** Volcano plots depict DEGs in OS tissues versus normal tissues across three GEO datasets (GSE12865, GSE14359, GSE36001). Red nodes denote upregulated genes (log2FC ≥ 1, *p* < 0.05), while green nodes indicate downregulated genes (log2FC ≤ -1, *p* < 0.05). **(D)** Venn diagram illustrates overlapping DEGs among the three GEO datasets.

The objective of this study is to elucidate the key molecular features of OS and the flowchart is shown in **Figure 1**. We selected three publicly available datasets from the GEO database (GSE12865, GSE14359, and GSE36001) and the statistical thresholds for DEGs were |log2FC|≥1 and p < 0.05. The results are as follows: 4,243 DEGs in GSE12865 (1,712 up-regulated, 2,531 down-regulated), 3,322 DEGs in GSE14359 (1,883 up-regulated, 1,439 down-regulated) and 890 DEGs in GSE36001 (271 up-regulated, 619 down-regulated) (**Figures 2A–C**). We then identified 159 DEGs overlapping in the three datasets through Venn diagrams (**Figure 2D**), which are potentially significant in the pathogenesis of OS. These genes may represent core molecular drivers of OS progression and require further validation.

### 3.2 Functional enrichment analysis of DEGs

To explore the biological context of the identified DEGs, we conducted GO and KEGG pathway enrichment analyses. These analyses revealed that DEGs were predominantly enriched in pathways related to tumor progression and immune regulation, including cell adhesion, apoptotic processes, extracellular matrix organization, and actin cytoskeleton remodeling (**Supplementary Figure S1**). These results provided useful biological insight and served as a foundation for downstream analysis. While such enrichment patterns are frequently observed in various cancer types, the findings supported the relevance of the selected DEGs to core tumor-associated processes and helped to guide subsequent hub gene identification.

### 3.3 Identification of hub genes via PPI network and module analysis

**Figure 3:**
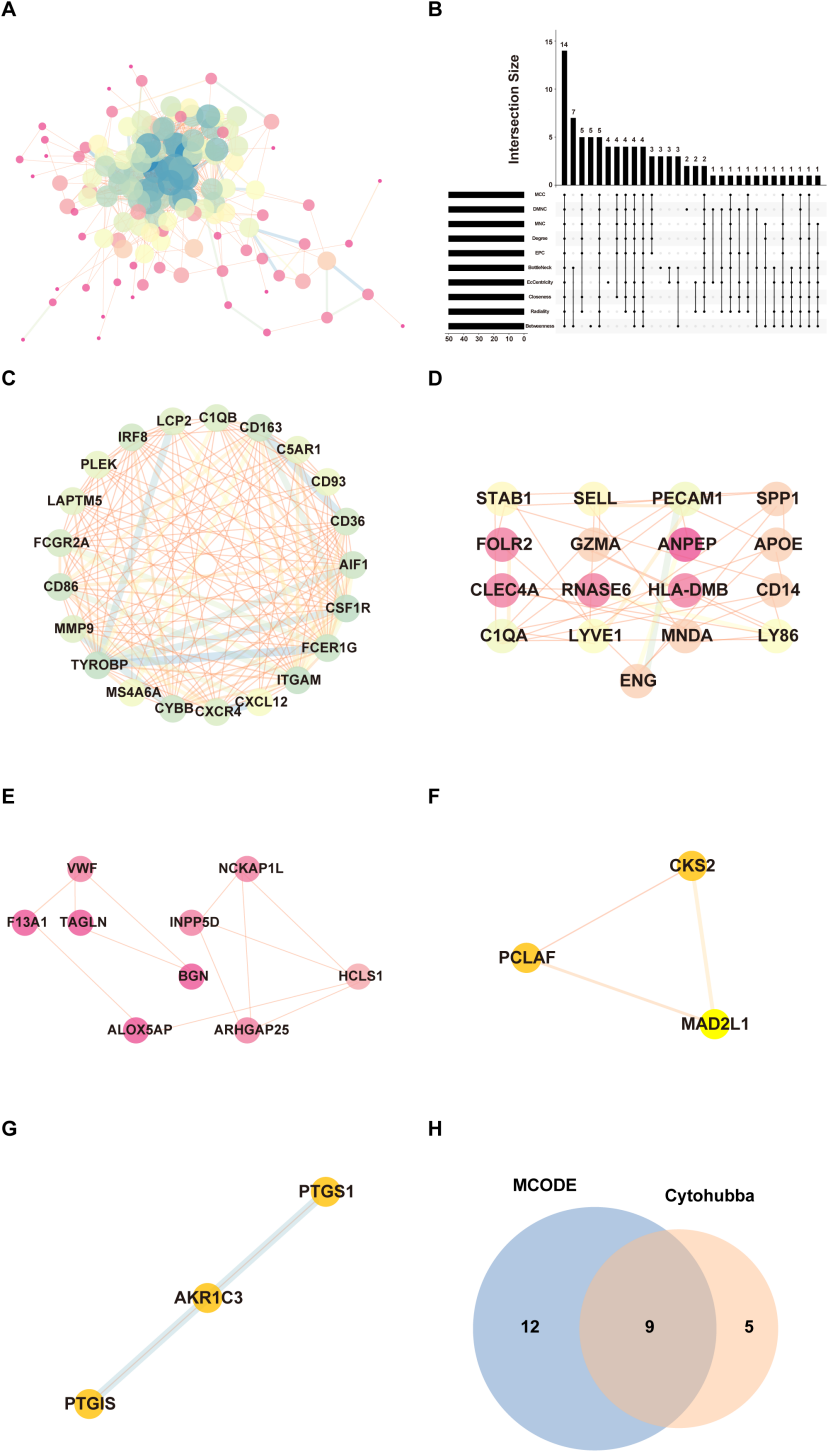
PPI networks and module analysis. **(A)** The PPI network of 159 DEGs. Each node represents a protein, while edges indicate protein-protein interactions. **(B)** Identification of hub genes by intersecting the top 50 genes from 10 algorithms using Cytohubba. **(C)** The highest-scoring core module identified through MCODE analysis, representing a subnetwork of genes with potential key functional roles. Nodes are highlighted to emphasize their significance. **(D–G)** Submodules extracted from the PPI network based on MCODE clustering. **(H)** Venn diagram showing the overlap between hub genes identified through MCODE and CytoHubba algorithm.

To explore protein interactions among identified DEGs, we built a PPI network with the STRING database and visualized it by Cytoscape software (**Figure 3A**). The network consisted of 134 nodes and 806 edges. This analysis then evaluated the interactions between these genes and identified 14 hub genes: C1QA, CD36, CD86, CXCL12, CXCR4, CYBB, ENG, FCER1G, GZMA, ITGAM, LAPTM5, PECAM1, PLEK and SELL (**Figure 3B**). The MCODE algorithm was applied in Cytoscape to detect densely connected modules, and five key modules were finally identified, of which the highest scoring module is shown in **Figure 3C**, consisting of 21 nodes and 153 edges. The other modules are shown in **Figures 3D–G**. The intersection results of MCODE and CytoHubba algorithm identified nine key hub genes: CD36, CD86, CXCL12, CXCR4, CYBB, FCER1G, ITGAM, LAPTM5, and PLEK (**Figure 3H**).

### 3.4 Elevated expression of hub genes in OS tissues

We assessed the expression levels of these nine hub genes in normal and OS tissues by data from TCGA and GTEx databases to identify their potential as prognostic genes. Eight genes (CD36, CD86, CXCR4, CYBB, FCER1G, ITGAM, LAPTM5, and PLEK) were observed upregulated in OS tissues, while CXCL12 was downregulated. Notably, the CXCL12-CXCR4 axis may affect tumor cell migration and the immune microenvironment (21). Elevated expression of CD36 and CYBB indicates their significant involvement in regulating lipid metabolism and oxidative stress during OS development (22, 23). The upregulation of immune-related genes such as CD86, FCER1G, and ITGAM suggests involvement of immune activation and inflammatory signaling within the osteosarcoma microenvironment. PLEK, which encodes a signaling adaptor protein, also showed a notable upregulation, meriting further investigation (**Supplementary Figure S2**). However, given the extremely limited number of normal control samples, the expression comparison based on public datasets should be regarded as exploratory in nature.

To further support the differential expression of hub genes between OS and normal tissues, we performed an additional expression analysis using our recently published OS transcriptomic dataset (24). Consistent with our observations from TCGA and GTEx, most hub genes—including PLEK, CYBB, CXCR4, and ITGAM—were significantly upregulated in osteosarcoma samples compared to normal controls (**Supplementary Figure S3**). These independent data reinforce the robustness of the expression trends and further support the relevance of these hub genes in the OS context.

### 3.5 Overall survival analysis of hub genes

**Figure 4:**
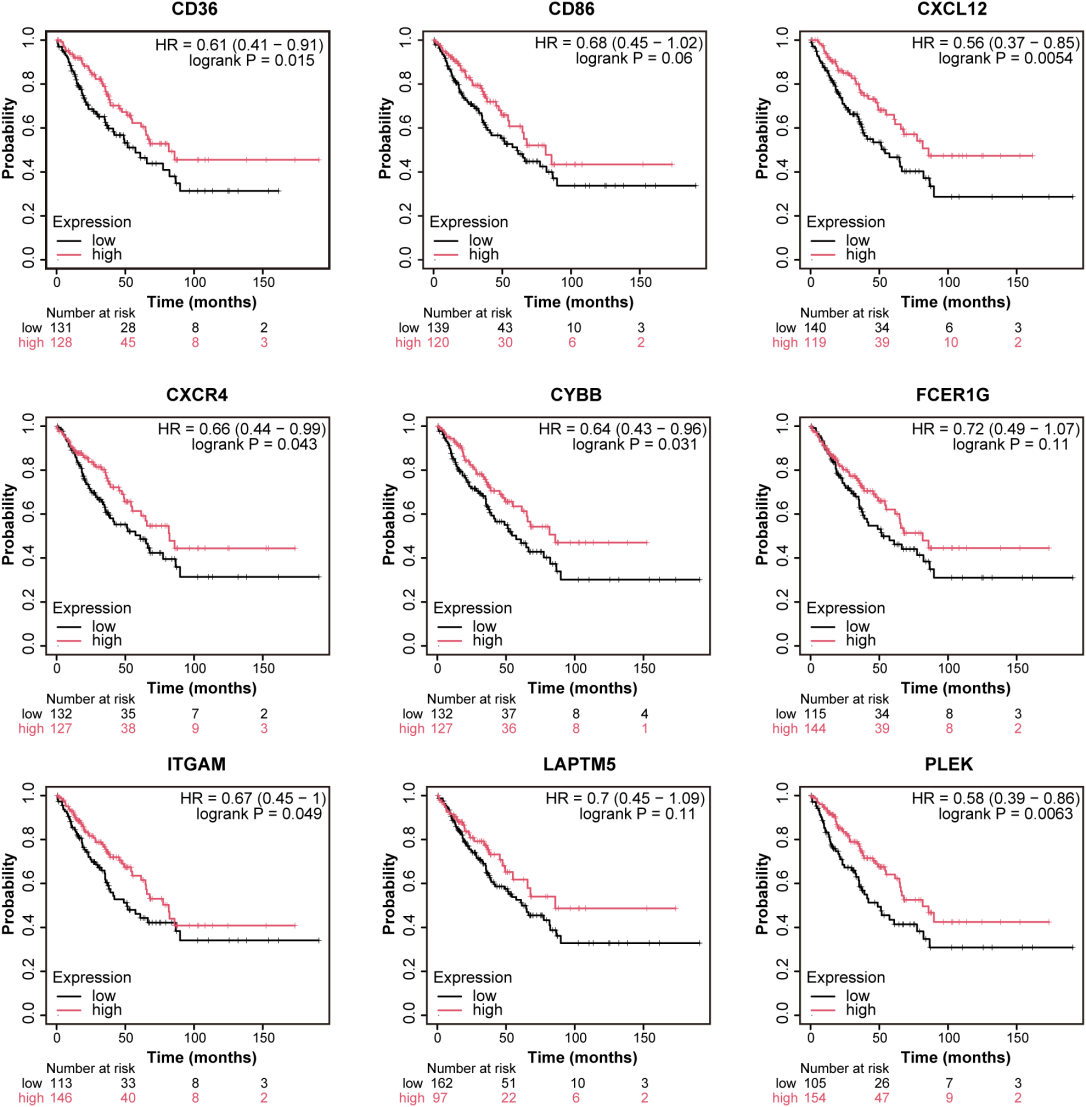
Prognostic validation of hub genes. Prognostic Validation of nine hub Genes Through Kaplan-Meier Survival Analysis. (CD36, CD86, CXCL12, CXCR4, CYBB, FCER1G, ITGAM, LAPTM5, PLEK) in OS using Kaplan-Meier (KM) plotter.

To evaluate the prognostic significance of these hub genes, we performed a Kaplan-Meier survival analysis. Six genes (CD36, CXCL12, CXCR4, CYBB, ITGAM, and PLEK) were significantly associated with overall survival in OS patients. Specifically, CD36 [HR = 0.61 (0.41-0.91), p = 0.015], CXCL12 [HR = 0.56 (0.37-0.85), p = 0.0054], CXCR4 [HR = 0.66 (0.44-0.99), p = 0.043], CYBB [HR = 0. 64 (0.43-0.96), p = 0.031], ITGAM [HR = 0.67 (0.45-1.00), p = 0.049], and PLEK [HR = 0.58 (0.39-0.86), p = 0.0063] were associated with significantly better prognosis (**Figure 4**). This highlights their potential as prognostic biomarkers and therapeutic targets for OS.

### 3.6 Genetic alterations in hub genes associated with OS

**Figure 5:**
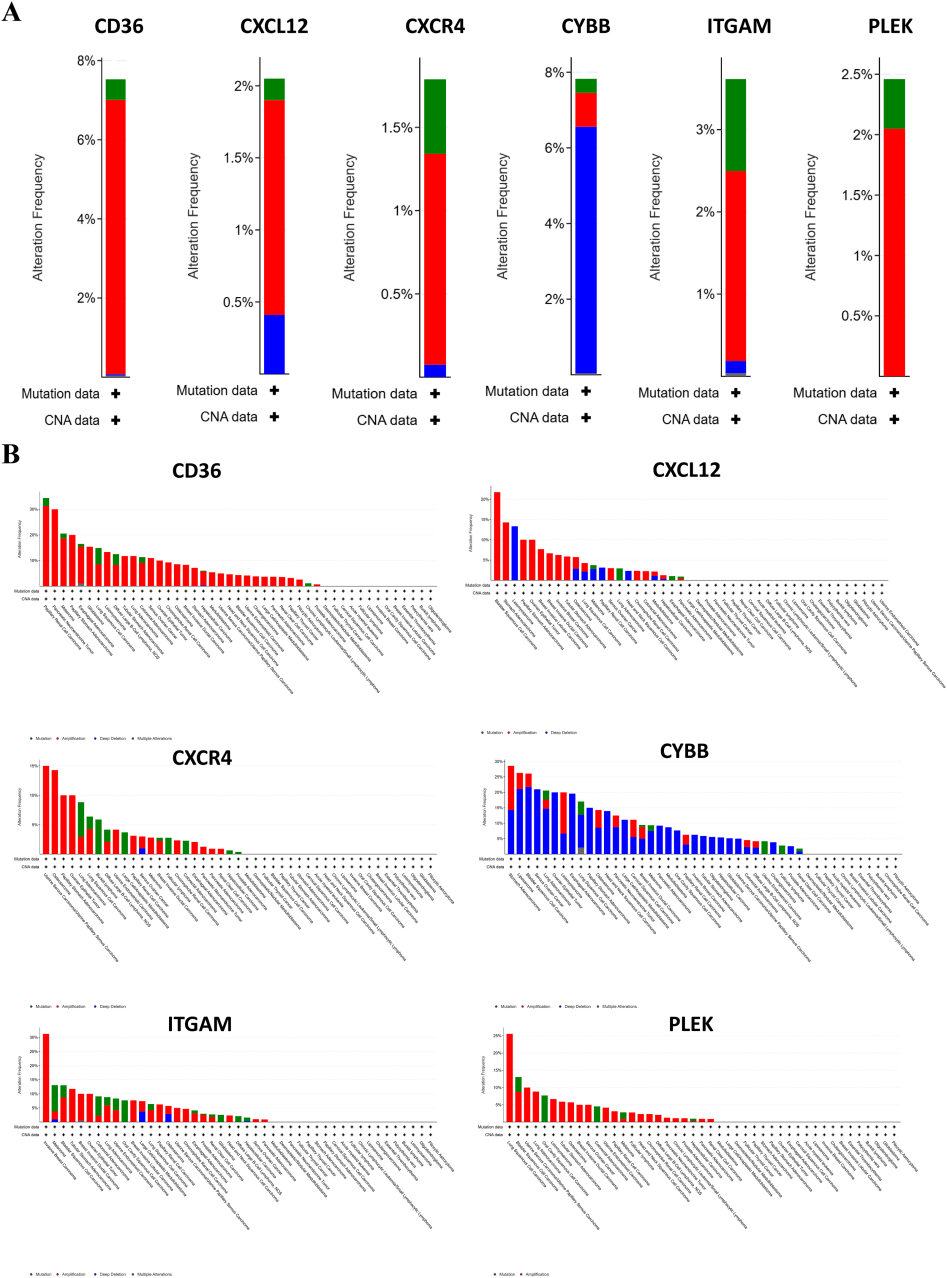
Genetic alterations of hub genes in pan-cancer. **(A)** Total genetic alteration frequencies of six hub genes (CD36, CXCL12, CXCR4, CYBB, ITGAM, PLEK) in pan-cancers, including mutations (green), copy number amplifications (red), and deletions (blue). **(B)** Distribution of genetic alterations in these genes across multiple cancer types.

The bar charts (**Figure 5A**) show the total alteration frequency of six hub genes (CD36, CXCL12, CXCR4, CYBB, ITGAM, PLEK) in pan-cancer. The most frequently altered genes were CD36 (7.88%) and CYBB (8.1%), mainly due to copy number amplification. Moderate alteration frequencies were observed in ITGAM and PLEK (3.63% and 2.53%), whereas lower frequencies were observed in CXCR4 and CXCL12 (1.87% and 2.14%). These findings suggest that variation in high-frequency genes is dominated by copy number amplification and that their overexpression may drive tumor-related functions. Bar charts (**Figure 5B**) showed the frequency and distribution of genetic alterations of these six genes in different cancer types. These hub genes showed different patterns of alterations in different tumors, suggesting their potential roles in a variety of cancer types except OS. Notably, CYBB showed a high frequency of deletions in various malignancies such as liposarcoma, bladder squamous cell carcinoma and severe ovarian cancer.

### 3.7 Relevance between hub genes and the immune microenvironment

**Figure 6:**
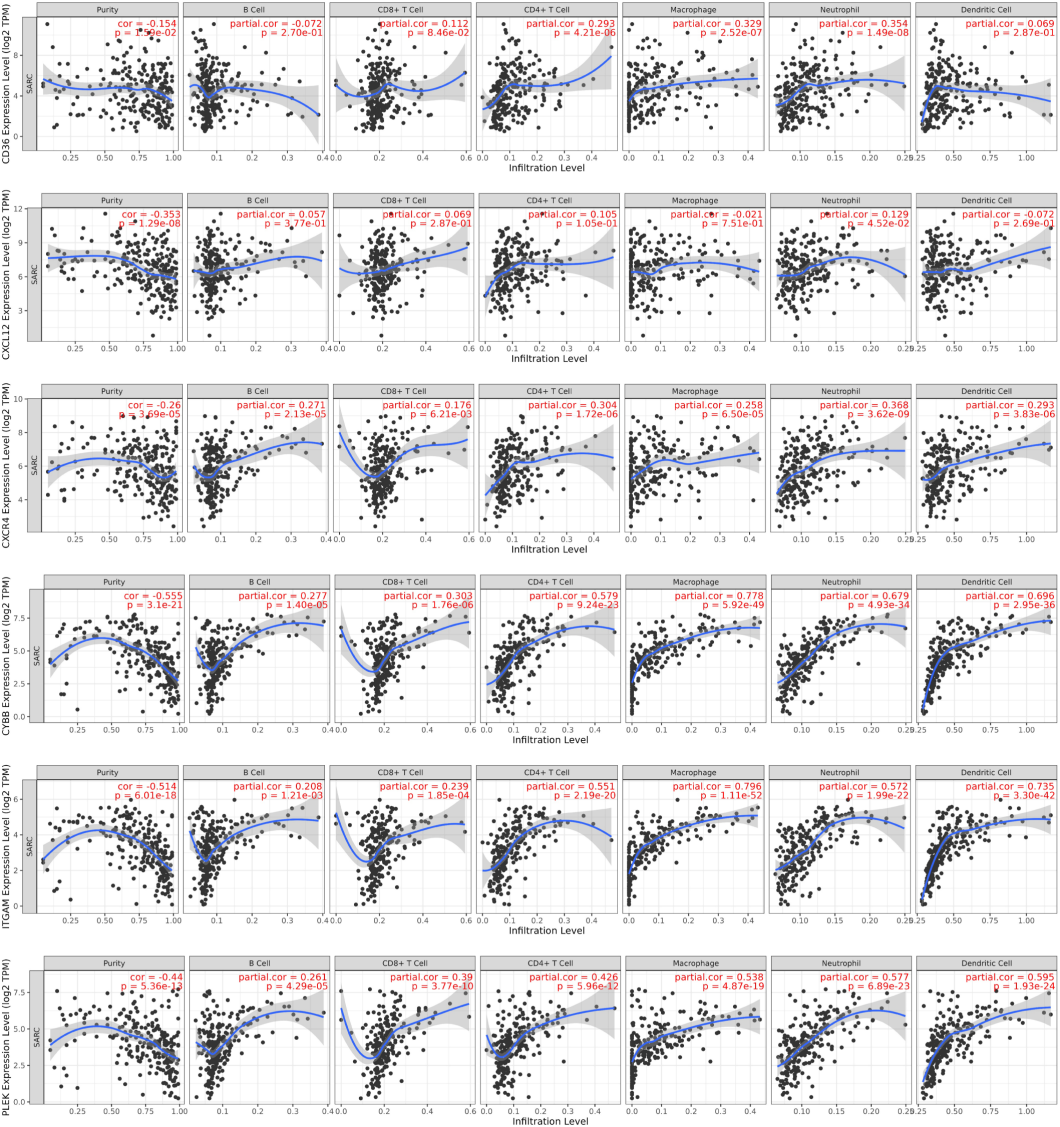
TIIC analysis of hub genes. Scatter plots depict the relationship between the expression levels of selected genes (log2 TPM) and immune infiltration levels across various immune cell types. Immune cell types are categorized as B cells, CD8+ T cells, CD4+ T cells, macrophages, neutrophils, and dendritic cells.

According to TIIC analysis (**Figure 6**), all six hub genes were negatively correlated with tumor purity (p < 0.001), which may indicate that these genes are closely associated with non-tumor cells like immune cells within TME. Among them, CYBB, ITGAM, and PLEK showed significantly negative correlations with tumor purity (ρ = -0.555, -0.514, and -0.44), suggesting their potential as markers of the immune microenvironment. CD36 exhibited a positive association with macrophages (ρ = 0.329) and neutrophils (ρ = 0.354), which may correspond to its involvement in lipid metabolism and immune modulation (25).

CXCL12 and CXCR4 are positively correlated with neutrophils (ρ = 0.129, 0.362), which may be related to their role in promoting neutrophil recruitment through chemotaxis (26). This indicates that the CXCL12-CXCR4 axis plays a role in promoting immune cell migration (27). The CXCL12-CXCR4 axis can recruit immune cells such as T cells and dendritic cells to TME, thereby enhancing immune surveillance and influencing tumor progression through immune-mediated pathways, consistent with the results of TIIC analysis in this study (28–31).

CYBB showed a positive correlation with macrophages (ρ = 0.778) and dendritic cells (ρ = 0.696), highlighting its central role in reactive oxygen species (ROS) generation in myeloid cells (32). It also showed the strongest negative correlation with tumor purity (ρ = -0.555), reflecting its potential central role in TME and immune regulation (33, 34).

ITGAM was strongly correlated with macrophages (ρ = 0.752), dendritic cells (ρ = 0.735), and CD4+ T-cells (ρ = 0.579), suggesting its involvement in immune cell adhesion and antigen immune processes of the presenting cells (35, 36).

Finally, PLEK was positively correlated with all immune cell types, highlighting its potential role in shaping the tumor immune landscape. Functionally, PLEK regulates actin cytoskeleton remodeling via integrins and Rac GTPases downstream of PKC. It is involved in leukocyte activation and integrin-mediated signaling, suggesting a role in macrophage recruitment and polarization. Its expression aligns with enhanced phagocytic and migratory activity in macrophages, neutrophils, and Tregs, supporting its contribution to immune cell activation and infiltration (37–39).

### 3.8 Functional validation, single-cell RNA sequencing and metabolic profiling.

**Figure 7:**
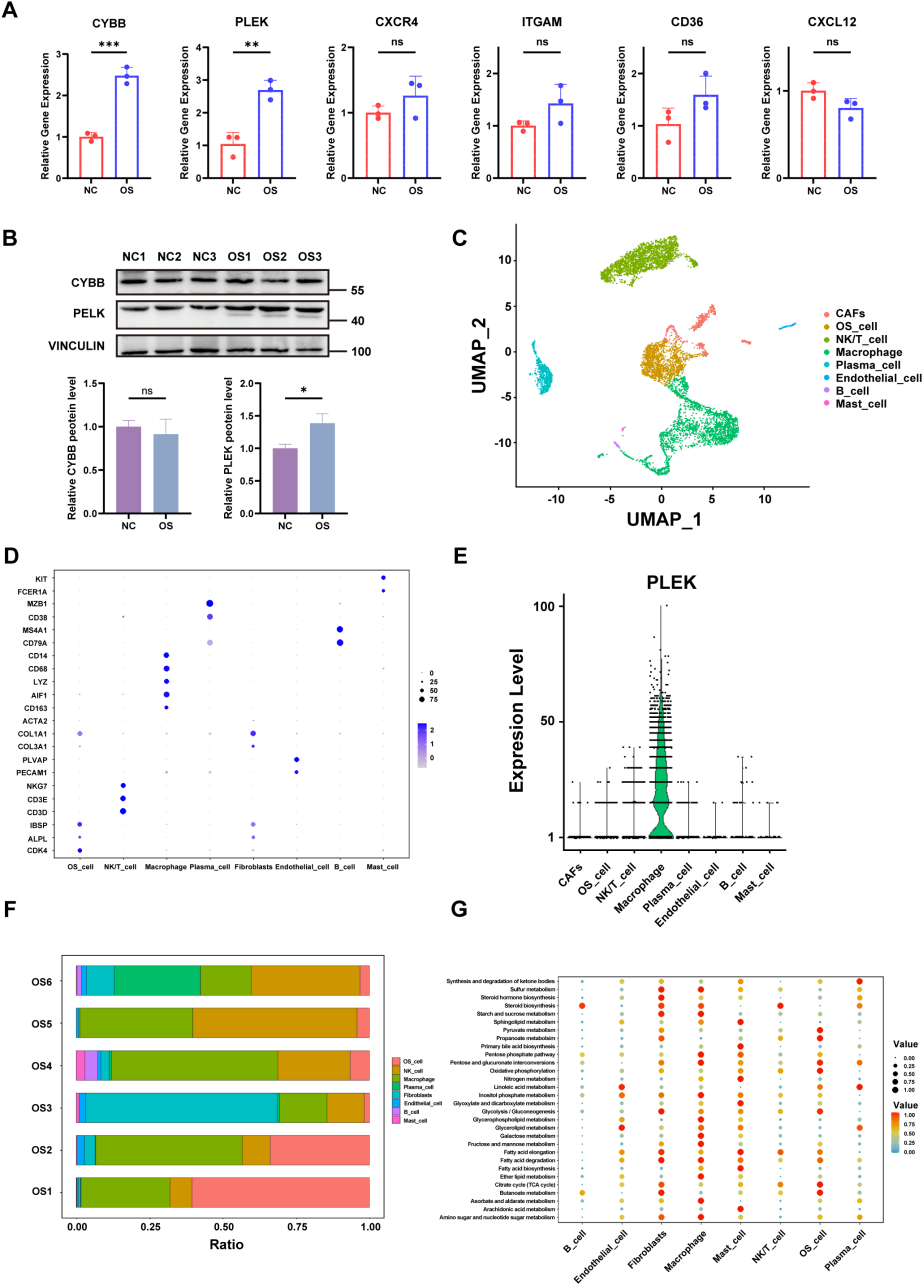
qRT-PCR, single-cell transcriptome and metabolic profiling. **(A)** The relative expression levels of the six hub genes in OS and normal tissues were detected by qRT-PCR. **(B)** Western blotting results for genes of interest. **(C)** UMAP Clustering and Cell-Type Identification in OS **(D)** Dot plot showing marker gene expression across OS cell types. **(E)** Violin plots showing expression levels of PLEK across cell types. **(F)** Proportional composition of cell types across six OS tissue samples. **(G)** Bubble plot depicting metabolic pathway enrichment analysis across OS cell types. p < 0.05 (*), p < 0.01 (**), p < 0.001 (***). “ns” denotes no statistical significance.

We validated the expression of six hub genes (CXCR4, CYBB, ITGAM, PLEK, CD36, and CXCL12) in OS and normal tissues respectively. The results of qRT-PCR showed that the expression of CYBB and PLEK was up-regulated in OS tissues compared with normal tissues, whereas there was no significant trend in the expression of CXCR4, ITGAM, CD36 and CXCL12 in both tissue types (P > 0.05) (**Figure 7A**).Then, we used Western blot analysis to detect the differences in protein expression levels of these two key genes in OS tissue and normal tissue. We found that the expression level of PLEK protein in OS tissue was higher than that in normal tissue, which was consistent with the PCR results, while CYBB protein expression showed no significant trend (**Figure 7B**).

Next, different cell populations were identified by single-cell RNA sequencing (scRNA-seq) in OS tissues, including cancer-associated fibroblasts (CAFs), OS cells, NK/T cells, macrophages, plasma cells, endothelial cells, B cells, and mast cells (**Figure 7C**). The expression of hub genes for each cell population was shown in **Figure 7D**. Violin plots showed that PLEK is predominantly expressed in macrophage subsets, with minimal expression in non-immune compartments, suggesting that this gene may be involved in the immune microenvironment of tumors, particularly in relation to immune function, immunosuppression, or pro-tumorigenic effects of macrophages (40) (**Figure 7E**).

Analysis of immune cell distribution in the six groups of OS tissues revealed a high percentage of macrophages in the tumor microenvironment, which was consistent with the identified hub genes expression patterns (**Figure 7F**). In addition, metabolic pathway enrichment analysis revealed that key pathways like glycolysis, oxidative phosphorylation and lipid metabolism were observed upregulated in macrophages (**Figure 7G**). And this metabolic reprogramming is also an important feature of cancer (41), for instance, reversing glycolysis to OXPHOS in cancer cells has been shown to induce cell death (42).

Among the nine hub genes identified through integrative bioinformatic analysis, PLEK was strategically prioritized for in-depth investigation based on a combination of biological and translational criteria. First, among all candidates, PLEK was the only gene consistently upregulated in osteosarcoma tissues at both the mRNA and protein levels, as confirmed by qRT-PCR and Western blotting. Second, single-cell RNA sequencing revealed that PLEK exhibited the highest cell-type specificity within macrophage subsets in the OS microenvironment, whereas other hub genes, such as CXCR4, were more broadly expressed across diverse immune cell populations. Third, PLEK showed one of the strongest associations with overall survival in OS patients, underscoring its prognostic relevance. Fourth, PLEK was enriched within metabolically active macrophage clusters characterized by elevated glycolytic and oxidative phosphorylation signatures, linking it directly to the metabolic axis of the tumor microenvironment—a key focus of this study. Collectively, these features place PLEK at the intersection of immune specificity, prognostic significance, and metabolic regulation, providing a robust rationale for its selection as a candidate for mechanistic exploration.

### 3.9 Interactions and communication between cells with TME of OS

**Figure 8:**
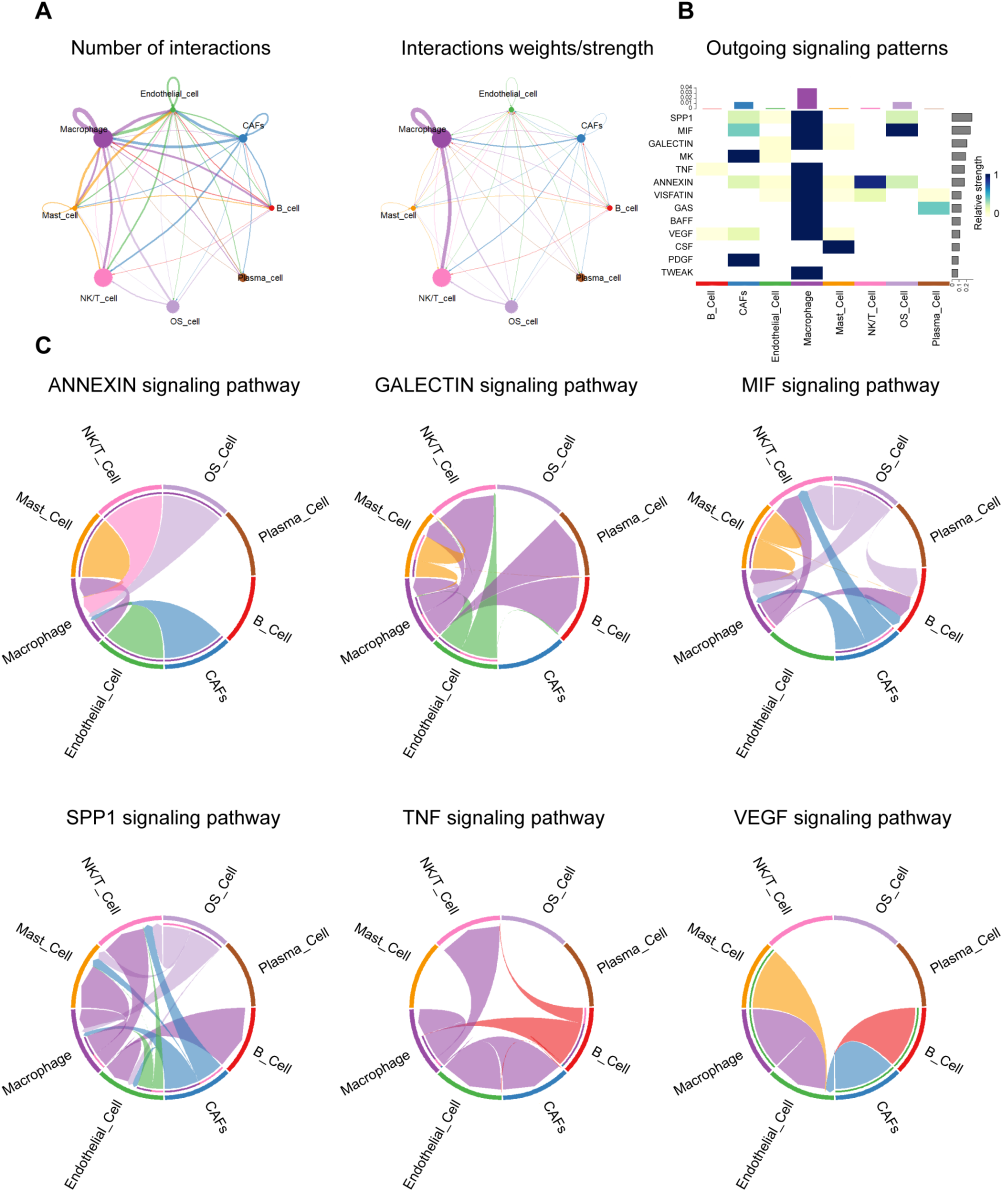
Cell-cell communication landscape. **(A)** Network diagram depicting the overall number (left) and strength (right) of cell-to-cell interactions between osteosarcoma-associated cell populations. **(B)** The heatmap responds to the efferent signaling pattern, showing the extent to which various types of cells act in different signaling axes. **(C)** Circle diagram showing the different intercellular crosstalk pathways in the osteosarcoma microenvironment mediated by ANNEXIN, GALECTIN, MIF, SPP1, TNF and VEGF signaling pathways.

To characterize the cellular communication network within the TME of OS, we performed a systematic ligand-receptor interaction analysis across OS cells and various stromal and immune populations. The global interaction landscape revealed that macrophages exhibited the highest number and strength of interactions with other cell types, most notably with OS cells (**Figure 8A**). This suggests that macrophages serve as central communication hubs within the TME and may play a pivotal role in shaping tumor behavior.

To dissect the signaling specificity driving these interactions, we next examined the outgoing signaling patterns of each cell type across key paracrine pathways (**Figure 8B**). Macrophages and OS cells emerged as dominant sources of multiple tumor-relevant signals, including SPP1, TNF, MIF, GALECTIN, ANNEXIN, and VEGF. These outgoing signals showed pathway-dependent specificity, reflecting diverse functional roles in modulating immune, stromal, and vascular responses. A more detailed analysis of pathway-specific interactions revealed distinct yet interconnected roles for each signaling axis (**Figure 8C**).

In the SPP1 pathway, macrophage-derived osteopontin (OPN) targeted OS cells, endothelial cells, and NK/T cells. This interaction aligns with OPN’s established roles in extracellular matrix remodeling, immune modulation, and metastatic niche formation. Encoded by SPP1, OPN reshapes the tumor immune microenvironment by inducing M2-like TAM polarization, suppressing dendritic cell migration, and promoting Th1 responses via IL-12 and TNF-α. OPN^+^ TAMs engage FAP^+^ CAFs through IL-1 and TGF-β signaling to restrict T cell infiltration and remodel the ECM, thereby limiting CTL access. In parallel, OPN enhances tumor growth, metastasis, and angiogenesis by activating survival and autophagy pathways, inducing EMT, and upregulating VEGF via integrin signaling (43).

TNF signaling, primarily from macrophages to OS cells and CAFs, points to a central role for inflammation in tumor-stroma interaction and possibly in stromal activation and immune suppression. TNFα and IL-1 inhibit osteogenic differentiation of OS cells by activating the ERK signaling pathway, thereby maintaining an undifferentiated state that facilitates tumor progression and metastasis. In the tumor microenvironment, macrophages are the primary source of TNFα (44).

MIF signaling was particularly robust between OS cells and macrophages, indicating a feedback loop wherein tumor-derived and macrophage-derived MIF mutually reinforce inflammatory signaling, angiogenesis, and macrophage recruitment. MIF is highly expressed in OS and promotes tumor proliferation and metastasis by activating the RAS/MAPK pathway, and is associated with poor prognosis. Targeting MIF can inhibit OS progression, enhance chemotherapy sensitivity, and exert anticancer effects by regulating the tumor microenvironment (45).

The GALECTIN pathway mediates bidirectional crosstalk among OS cells, macrophages, and CAFs. Galectins, a conserved family of glycan-binding proteins, regulate tumor progression by integrating intracellular and extracellular signals in both cancer and stromal compartments (46). Galectin-1 serves as a diagnostic marker distinguishing chondroblastic OS from conventional chondrosarcoma (47), and has also been linked to OS progression and metastasis (48). Galectin-3 promotes OS malignancy via a triple mechanism: driving bone resorption through RANKL/M-CSF signaling, enhancing metastasis via FAK/Src/β-catenin activation, and conferring chemoresistance by inhibiting apoptosis via its NWGR motif (49). Galectin-9, through interaction with Tim-3, modulates tumor immunity; blockade of the Gal-9/Tim-3 axis elicits anti-tumor immune responses and suppresses tumor growth (50).

In the ANNEXIN pathway, macrophages initiate key signals to OS cells, CAFs, and endothelial cells, highlighting annexin-mediated roles in inflammation resolution and membrane repair within the tumor microenvironment. AnxA1, abundantly expressed in macrophages, suppresses inflammation via formyl peptide receptors (FPRs), shaping the immune milieu. In OS cells, enhanced AnxA2 translocation to the plasma membrane releases TFEB from the AnxA2-TFEB complex, promoting autophagy and regulating differentiation. Additionally, AnxA2 enhances membrane repair and facilitates OS metastasis. Elevated ANXA3 expression further promotes OS cell proliferation, migration, and invasion (51–53).

Interestingly, VEGF signaling originated mainly from OS cells and was directed toward endothelial and immune cells, aligning with its classical role in promoting angiogenesis and vascular remodeling. Angiogenesis critically influences tumor growth and metastatic potential, and is recognized as one of the six hallmarks of cancer (54). Vascular endothelial growth factor (VEGF), a central mediator of angiogenesis and vasculogenesis, plays a pivotal role in the molecular pathogenesis of tumor progression and metastasis (54). Targeting VEGF signaling has been shown to suppress osteosarcoma cell proliferation and induce apoptosis (55).

Collectively, these findings reveal a complex, multi-pathway signaling network orchestrated by OS cells and macrophages, characterized by both unidirectional and reciprocal communication. This network underpins key hallmarks of OS progression, including chronic inflammation, stromal activation, immunomodulation, and angiogenesis. Macrophages, in particular, emerge as key amplifiers and mediators of tumor-promoting signals, highlighting their potential as targets for therapeutic intervention.

### 3.10 PLEK knockdown facilitates tumor-promoting behavior of OS cells in a macrophage-influenced microenvironment

**Figure 9:**
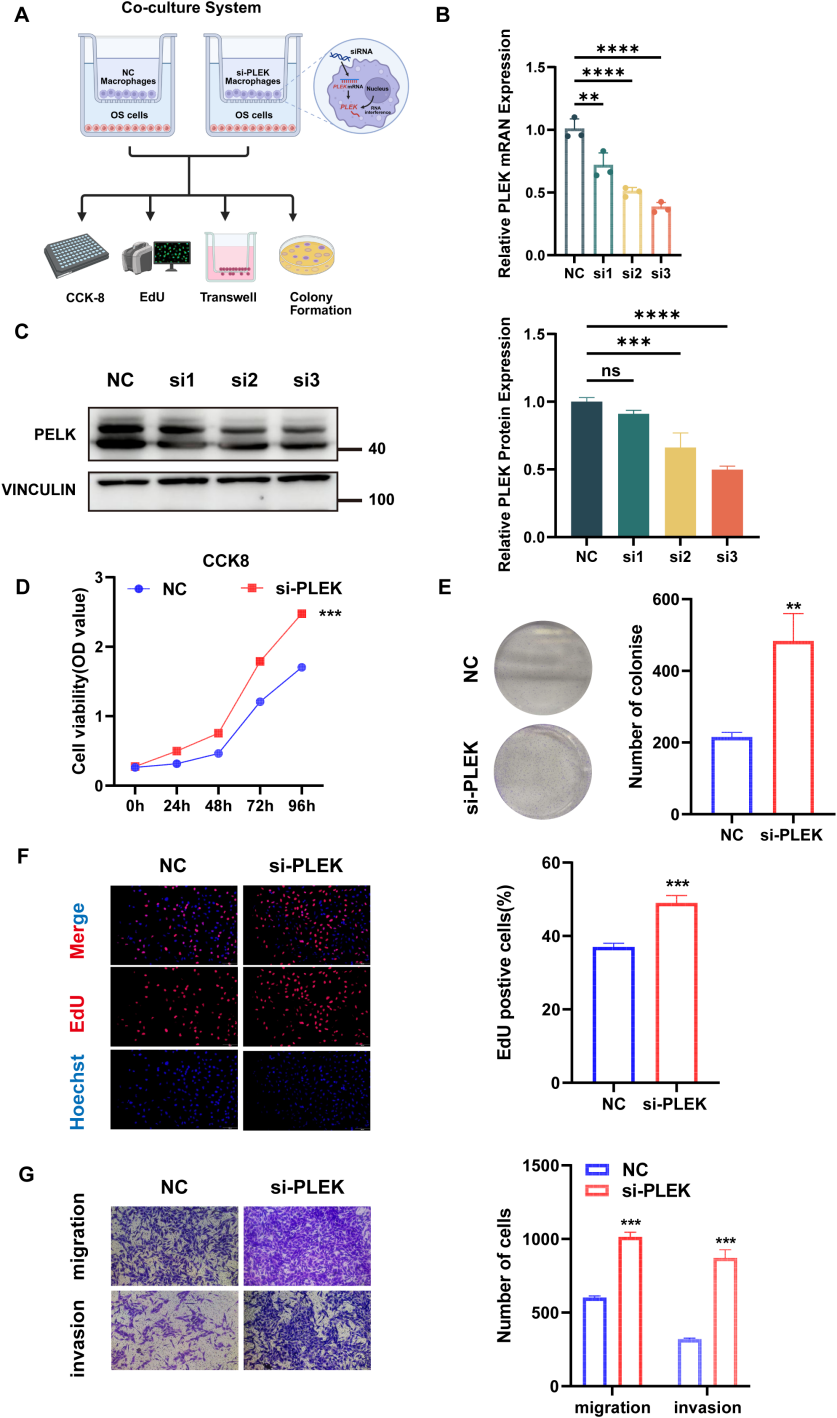
Subsequent validation of the hub gene PLEK. **(A)** Schematic diagram of the transfection co-culture system and downstream functional assays. **(B)** Quantitative PCR confirmed the efficiency of PLEK knockdown at the mRNA level in OS cells following siRNA transfection. **(C)** Western blot analysis validated the expression levels of PLEK protein after siRNA transfection. **(D)** Cell survival rates of the NC and si-PLEK groups detected by CCK-8 at multiple time points. **(E)** Representative images and quantification of colony formation assay in NC and si-PLEK groups. **(F)** The results of EdU uptake experiments in the NC and si-PLEK groups were quantified by determining the percentage of EdU-positive cells. **(G)** Representative images and quantitative results of migration and invasion of osteosarcoma cells in the NC and si-PLEK groups, using the Transwell assay.

Based on previous studies demonstrating extensive intercellular signaling between macrophages and OS cells, particularly through pro-tumor signaling pathways such as MIF, SPP1, and TNF, we aimed to functionally assess the contribution of the cytoskeletal adaptor protein PLEK (associated with immune cell signaling) to the malignant behavior of OS cells. To this end, we established a Transwell-based co-culture system to mimic the interactions between macrophages and OS cells under paracrine conditions in TME. OS cells were co-cultured with macrophages transfected with control siRNA (NC) or PLEK-specific siRNA (si-PLEK), as shown in **Figure 9A**. This allowed us to conduct a controlled assessment of PLEK function in a tumor-supportive cytokine environment. First, we confirmed the successful knockdown of PLEK by transfecting macrophages with three independent siRNAs. qPCR results showed that PLEK mRNA expression levels were significantly downregulated in the siRNA groups, particularly in the si2 and si3 groups (**Figure 9B**), and corresponding PLEK protein expression levels were also significantly reduced (**Figure 9C**).

To assess cell proliferation, we performed a 96-hour CCK-8 assay. Compared with the NC group, the survival rate of OS cells in the si-PLEK group was significantly higher, with differences becoming evident after 48 hours and further amplified at 72 and 96 hours (**Figure 9D**). Subsequently, we assessed the clonogenic capacity of OS cells via colony formation assays. The number and volume of colonies formed by si-PLEK group cells were higher than those in the NC group (**Figure 9E**), consistent with the enhanced long-term proliferative capacity. These results were validated by EdU uptake experiments, showing that the percentage of EdU-positive (proliferating) nuclei in PLEK-silenced OS cells was significantly higher (**Figure 9F**), indicating more active DNA synthesis and cell cycle progression. To investigate whether PLEK influences the migration and invasion capabilities of OS cells (key determinants of metastatic potential), we conducted Transwell migration assays. PLEK knockdown significantly promoted cell migration and invasion (**Figure 9G**), with a significant increase in the number of cells that migrated through the membrane in the si-PLEK group. These results collectively indicate that PLEK inhibits the malignant transformation of OS cells by regulating multiple aspects, including proliferation, clonogenic survival, and invasiveness. Mechanistically, PLEK may exert its effects through macrophage-derived cytokine signaling pathways, such as the MIF and TNF pathways previously identified as active in macrophage-osteosarcoma cell communication. As a cytoskeletal regulator, PLEK may integrate upstream inflammatory signals to promote cytoskeletal remodeling and cell migration. Importantly, this study provides functional evidence that disrupting PLEK expression in OS cells enhances the tumor-promoting effects of macrophage-derived paracrine signals. These findings suggest that PLEK may represent a potential therapeutic target, potentially linking signals from the immune microenvironment to the malignant progression of OS.

## 4 Discussion

OS, the most common primary malignant bone tumor in adolescents and young adults, is characterized by aggressive growth, early metastasis, and dismal outcomes despite multimodal treatments encompassing surgical resection and cytotoxic chemotherapy. While advances in genomics and imaging have deepened our understanding of OS biology, survival rates for patients with recurrent or metastatic disease remain low (1, 2, 56). Emerging evidence suggests that tumor progression and therapeutic resistance are shaped not only by intrinsic genetic alterations but also by the TME, wherein immunosuppressive cues and metabolic reprogramming collectively foster an immune-evasive niche (3, 4).

In this study, we employed integrative bioinformatic, bulk transcriptomic, and single-cell RNA sequencing approaches to delineate key immune-metabolic regulators in OS. We identified pleckstrin (PLEK)—a pleckstrin homology (PH) domain-containing cytoskeletal adaptor protein—as a hub gene predominantly expressed in macrophage subsets within OS tissues. Notably, high PLEK expression was associated with improved overall survival and increased infiltration of immune cells, particularly macrophages and dendritic cells, suggesting its role in sustaining a metabolically active, immune-stimulatory TME. Functional assays confirmed that PLEK knockdown in macrophages enhanced OS cell proliferation, migration, invasion, and clonogenic potential, underscoring its tumor-suppressive function via macrophage-mediated crosstalk.

Although pleckstrin family members such as PLEK2 and CNK1 have been implicated in tumor progression through PI3K-Akt signaling and KRAS-mediated immune modulation (38, 57–59), the role of classical PLEK (Plek1) in solid tumors remains largely unexplored. Initially characterized as a protein kinase C substrate in platelets and leukocytes (60, 61), PLEK has garnered limited attention in oncology. Here, we present the first comprehensive evidence that PLEK expression in tumor-infiltrating macrophages contributes to anti-tumor immunity in OS, potentially by reprogramming metabolic and signaling networks within the TME.

The TME of OS is profoundly immunosuppressive, limiting the efficacy of immune-based therapies (11). The infiltration of immune cells, particularly tumor-associated macrophages (TAMs), is a hallmark of OS microarchitecture, and its heterogeneity and plasticity make them key coordinators in the tumor microenvironment (40, 62). M1-like macrophages promote anti-tumor immunity via proinflammatory cytokines, while M2-like macrophages support tumor growth, angiogenesis, and immune escape (63). Single-cell transcriptomic analysis revealed that PLEK is predominantly expressed in a subset of tumor-infiltrating macrophages with elevated metabolic signatures, including increased glycolysis and oxidative phosphorylation. These cells appear to exhibit a distinct phenotype beyond the conventional M1/M2 framework. The enrichment of PLEK in metabolically active macrophages, together with the observation that PLEK expression was associated with reduced OS cell proliferation, migration, and invasion, suggests that these macrophages may play a supportive role in anti-tumor responses. This is in line with recent studies indicating that the metabolic state of macrophages may influence their functional polarization, and that enhanced mitochondrial metabolism could potentially contribute to tumor-restraining immune activity (64).

Intercellular communication within the TME further underscores the significance of PLEK-expressing macrophages. Our ligand–receptor interaction analysis highlighted extensive crosstalk between macrophages and OS cells through key immunoregulatory pathways. These interactions regulate a wide range of biological processes, from angiogenesis and immune suppression to ECM remodeling and autophagy. The observed enhancement of OS malignancy following PLEK knockdown may be attributed, at least in part, to dysregulation of these signaling pathways and the resulting shift in macrophage function toward a tumor-promoting phenotype.

In parallel with immune remodeling, metabolic reprogramming is now established as a hallmark of cancer, enabling tumor cells to meet bioenergetic and biosynthetic demands under hypoxic and nutrient-limited conditions (65). OS cells, like many aggressive malignancies, exhibit enhanced glycolysis (Warburg effect) despite oxygen sufficiency, supporting rapid growth and immune evasion (66). However, this metabolic shift is not confined to tumor cells; recent studies reveal that immune and stromal components within TME also undergo metabolic adaptation, which in turn influences their phenotype and function (67). Our findings that PLEK expression marks metabolically active macrophage clusters and suppresses OS malignancy suggest a novel role for PLEK in regulating immune cell metabolism to sustain anti-tumor immunity. Indeed, therapeutic strategies targeting metabolic checkpoints in immune cells—such as activation of oxidative phosphorylation or inhibition of fatty acid oxidation—have shown promise in preclinical models of solid tumors (68).

From a therapeutic standpoint, modulating the immune-metabolic axis of the OS microenvironment represents a promising strategy to overcome current treatment limitations. OS is widely recognized as an immunologically “cold” tumor, characterized by low T-cell infiltration and a suppressive TME, which may explain the poor clinical response to immune checkpoint inhibitors when used alone (56). To address this, recent preclinical studies have explored combination approaches that aim to remodel the TME—such as eliminating immunosuppressive M2-like macrophages, reprogramming TAM metabolism, or enhancing the recruitment and activation of cytotoxic T cells—and have shown synergistic anti-tumor effects (69, 70). Our findings position PLEK as a potential target within this immunometabolic framework. By regulating both macrophage metabolism and immune activation, PLEK may serve as a molecular switch that reshapes the TME from a tumor-permissive to a tumor-restrictive state. Therapeutically enhancing PLEK activity, or mimicking its downstream effects, could promote the emergence of metabolically active, immunostimulatory macrophages—thereby creating conditions that are more favorable for immunotherapy to be effective.

Nonetheless, several questions remain. The mechanistic pathways linking PLEK to macrophage polarization and metabolic rewiring warrant further investigation. It remains to be determined whether PLEK directly modulates metabolic enzyme expression or acts via upstream signaling nodes such as PI3K-AKT or mTOR, both of which govern immune cell metabolism and function. Additionally, while our study primarily focused on the role of PLEK in macrophages, its potential functions in other immune or stromal cell subsets remain to be elucidated. Furthermore, clinical validation in larger, multi-center OS cohorts, as well as *in vivo* modeling of PLEK-targeted interventions, will be critical to assess its translational relevance and therapeutic potential.

In conclusion, this study identifies PLEK as a novel immune-metabolic modulator in OS. PLEK expression in tumor-infiltrating macrophages is associated with favorable prognosis, increased immune infiltration, and a metabolically active TME that suppresses OS malignancy. Functional *in vitro* assays confirm PLEK’s tumor-suppressive role in macrophage–OS interactions. Our results underscore the importance of immune-metabolic crosstalk in OS progression and highlight PLEK as a promising biomarker and therapeutic target. Future work delineating the regulatory circuitry of PLEK and its downstream effectors may yield new avenues for immunometabolic therapy in OS and beyond.

## Data Availability

The datasets presented in this study can be found in online repositories. The names of the repository/repositories and accession number(s) can be found in the article/**Supplementary Material**.

## Supplementary material

Supplementary Figure 1 Functional Enrichment Analysis of DEGs including KEGG and GO analyses.

Supplementary Figure 2 Expression Levels of Hub Genes in OS and Normal Tissues.

Supplementary Figure 3 Expression heatmap of nine hub genes in OS and normal tissues based on our independent transcriptomic dataset.

Supplementary Figure 4 Violin plots showing expression levels of all hub genes across cell types.

Supplementary Figure 5 Clustering and quality control of GSE162454 single cells. **(A)** Cells filtered based on mitochondrial and erythrocyte gene content. **(B)** Top 3,000 variable genes highlighted (red); top 10 labeled.
