# Supplementary figures and images for: The osteosarcoma immune microenvironment in progression: PLEK as a prognostic biomarker and therapeutic target
Source: Front Immunol. 2025 Aug 15;16:1651858. doi: 10.3389/fimmu.2025.1651858 (PMC12394983; doi:10.3389/fimmu.2025.1651858)

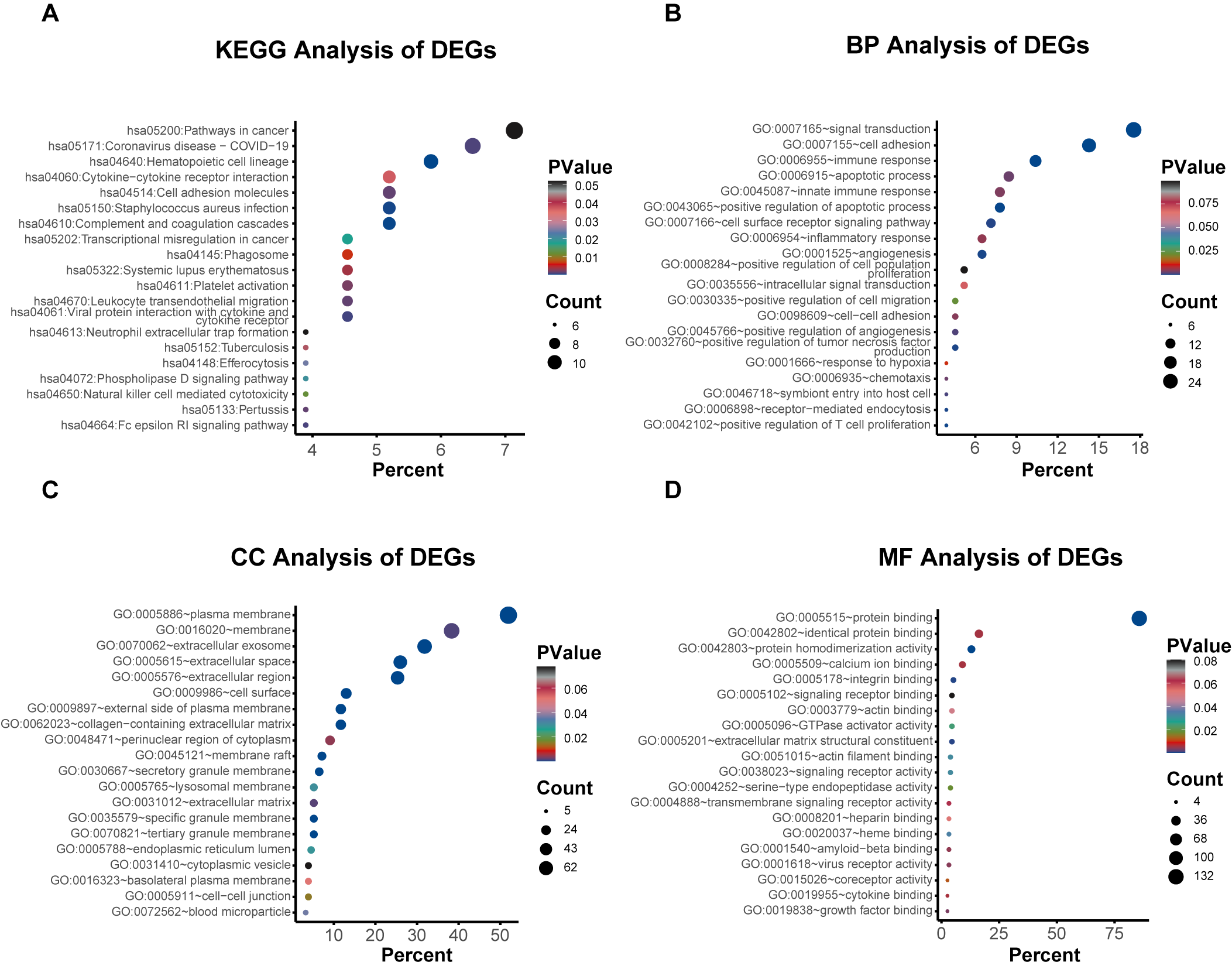

Supplement: Supplementary Figure 1 — Functional Enrichment Analysis of DEGs including KEGG and GO analyses. [file Image1.tif]

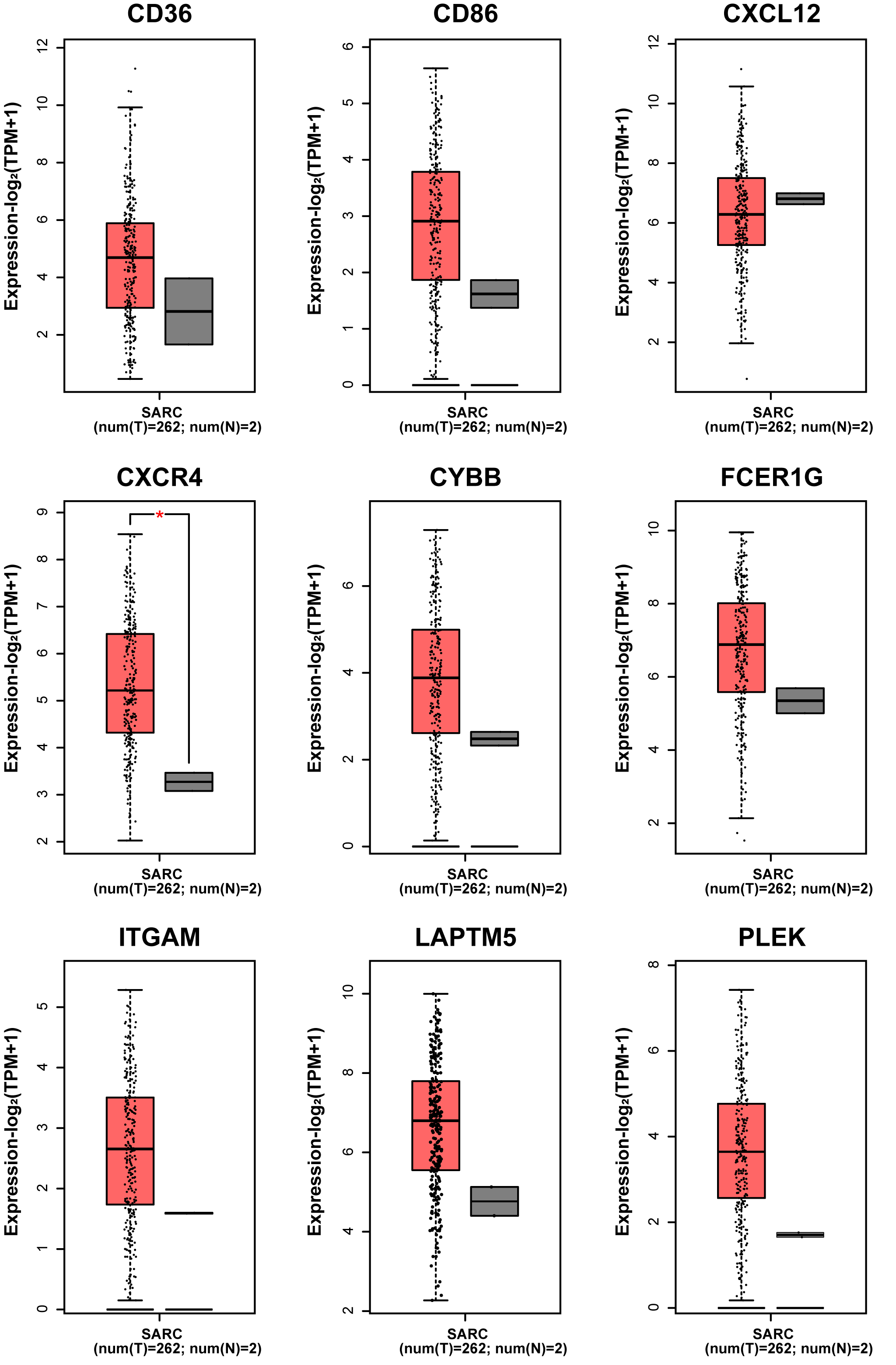

Supplement: Supplementary Figure 2 — Expression Levels of Hub Genes in OS and Normal Tissues. [file Image2.tif]

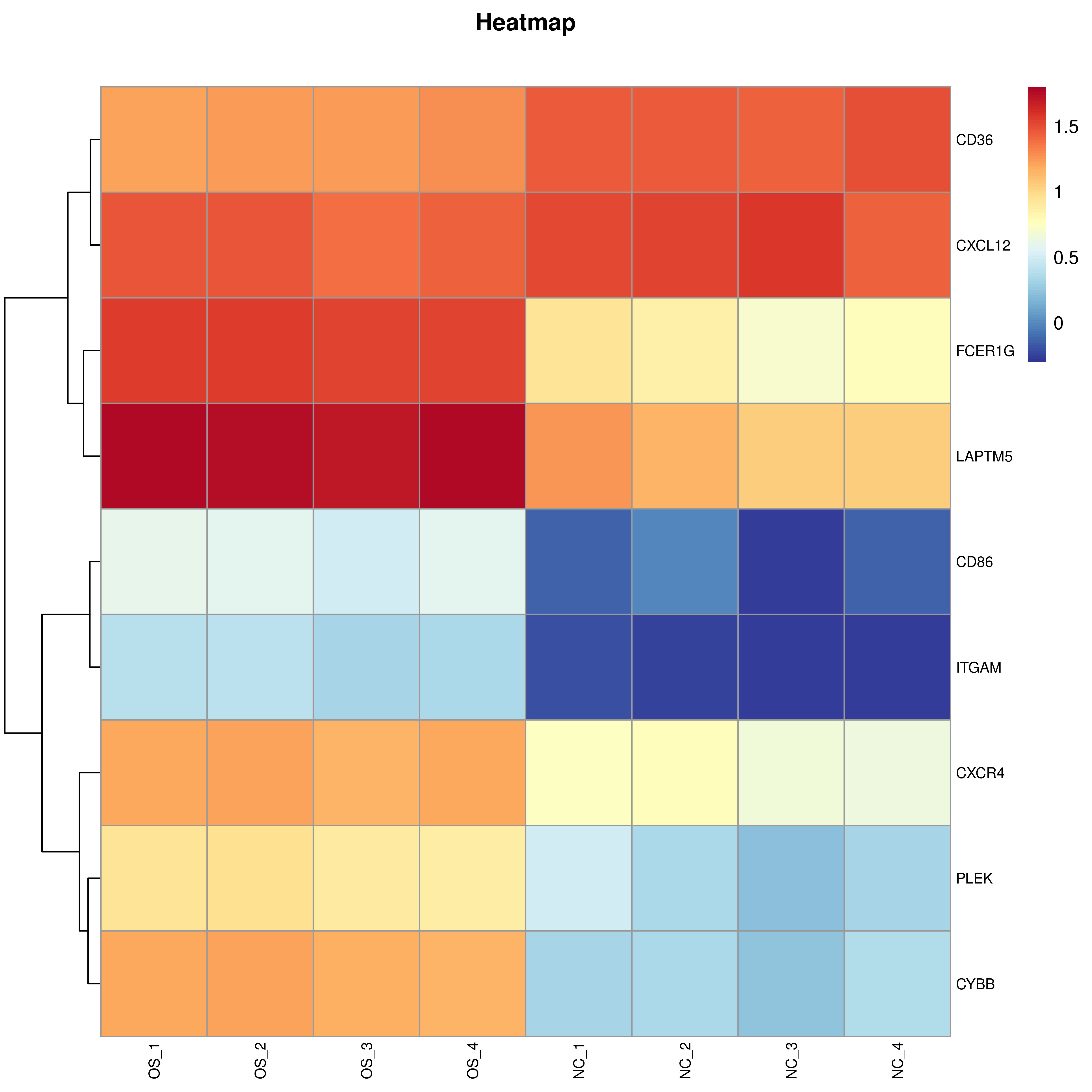

Supplement: Supplementary Figure 3 — Expression heatmap of nine hub genes in OS and normal tissues based on our independent transcriptomic dataset. [file Image3.tif]

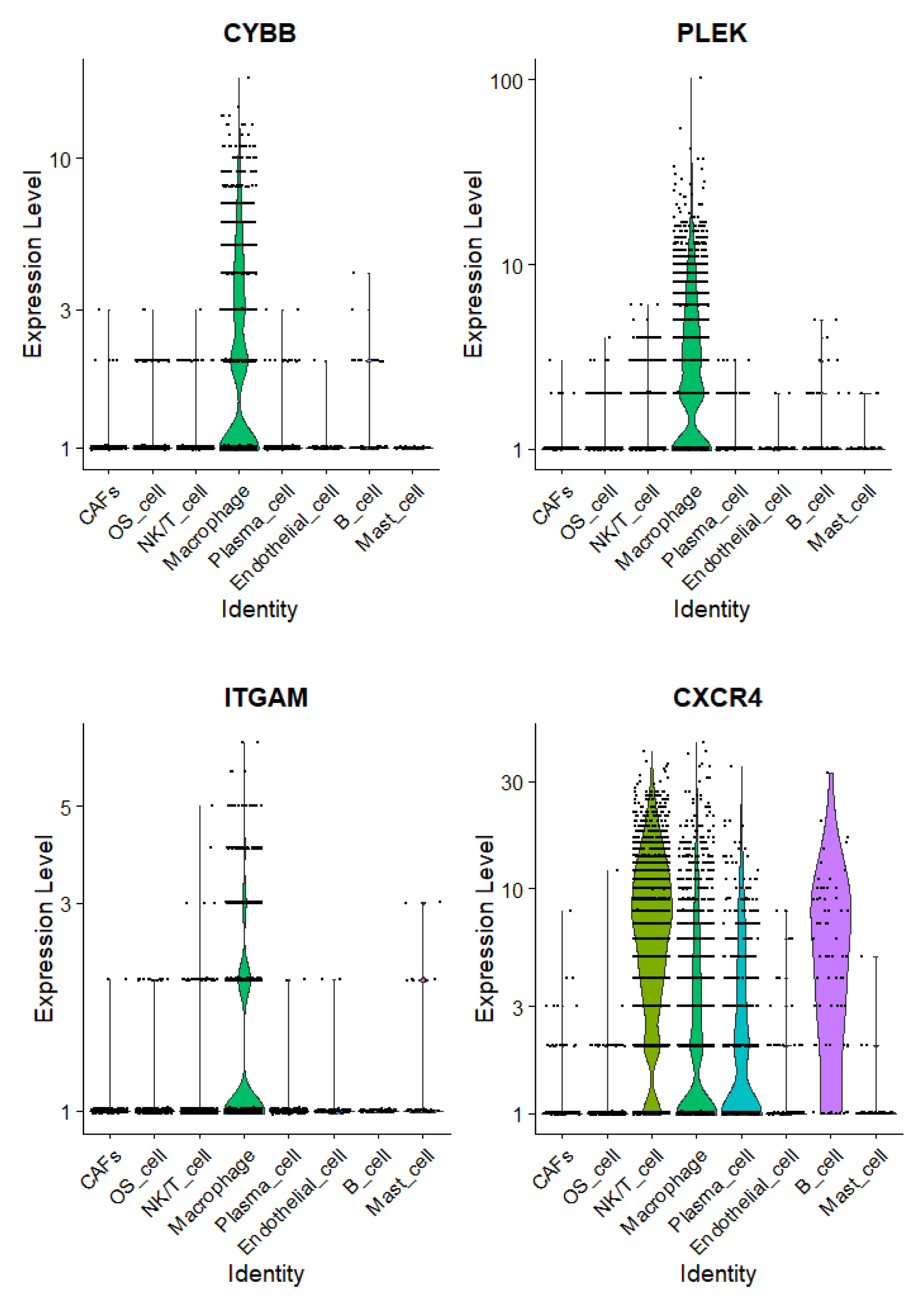

Supplement: Supplementary Figure 4 — Violin plots showing expression levels of all hub genes across cell types. [file Image4.tif]

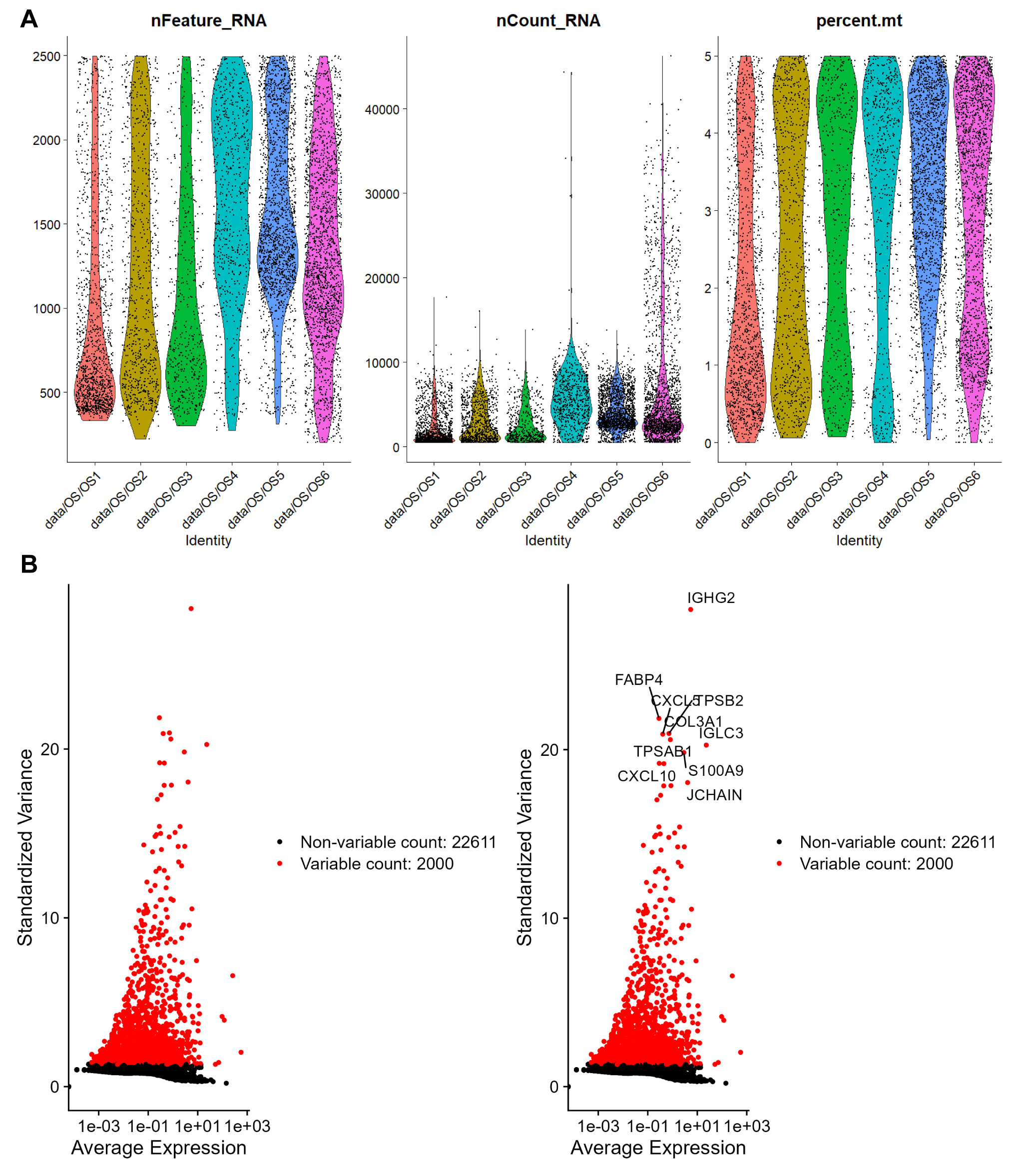

Supplement: Supplementary Figure 5 — Clustering and quality control of GSE162454 single cells. (A) Cells filtered based on mitochondrial and erythrocyte gene content. (B) Top 3,000 variable genes highlighted (red); top 10 labeled. [file Image5.tif]

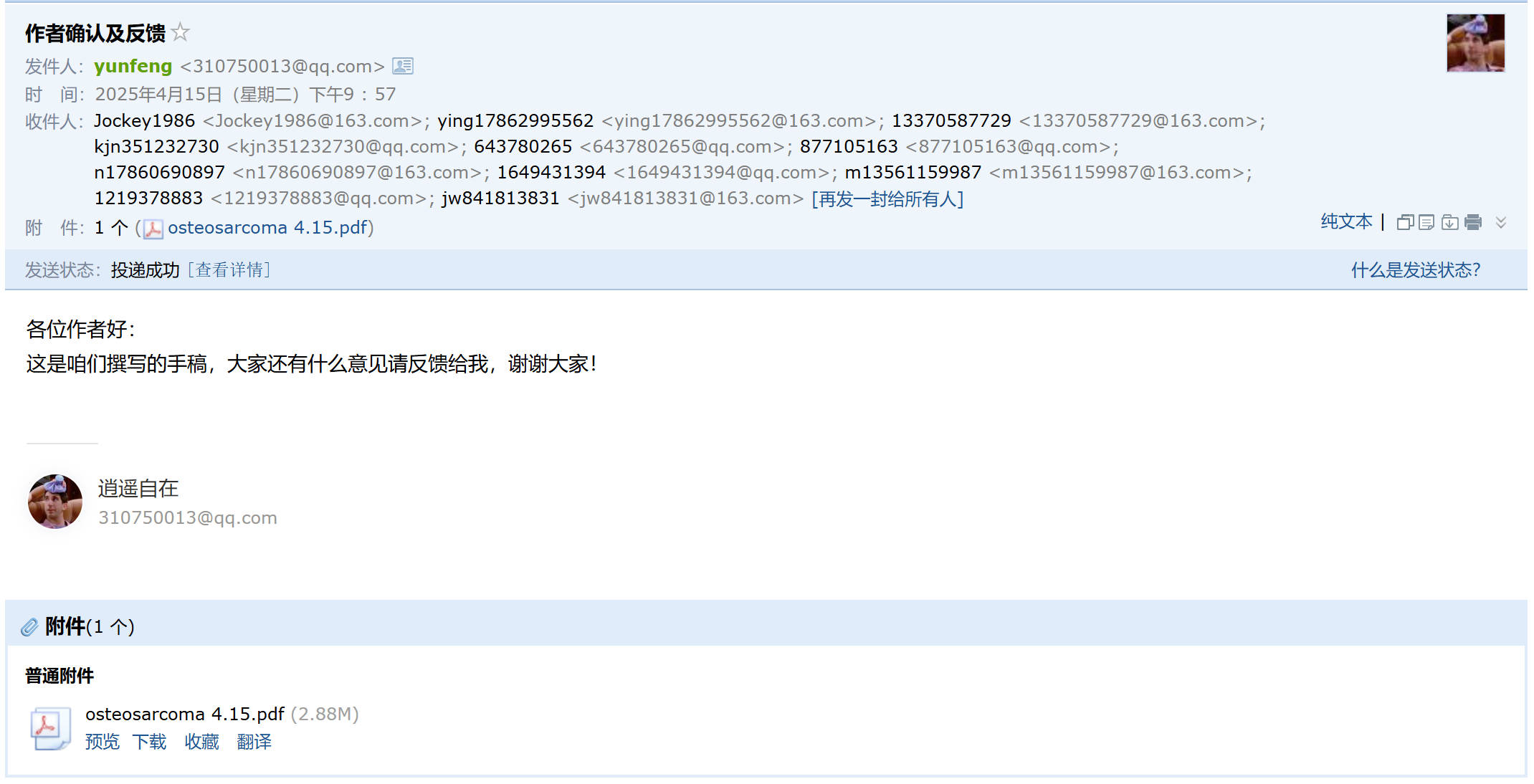

Supplement: Supplementary file 6 [file DataSheet1.zip › Authors and contributors/ALL.jpg]

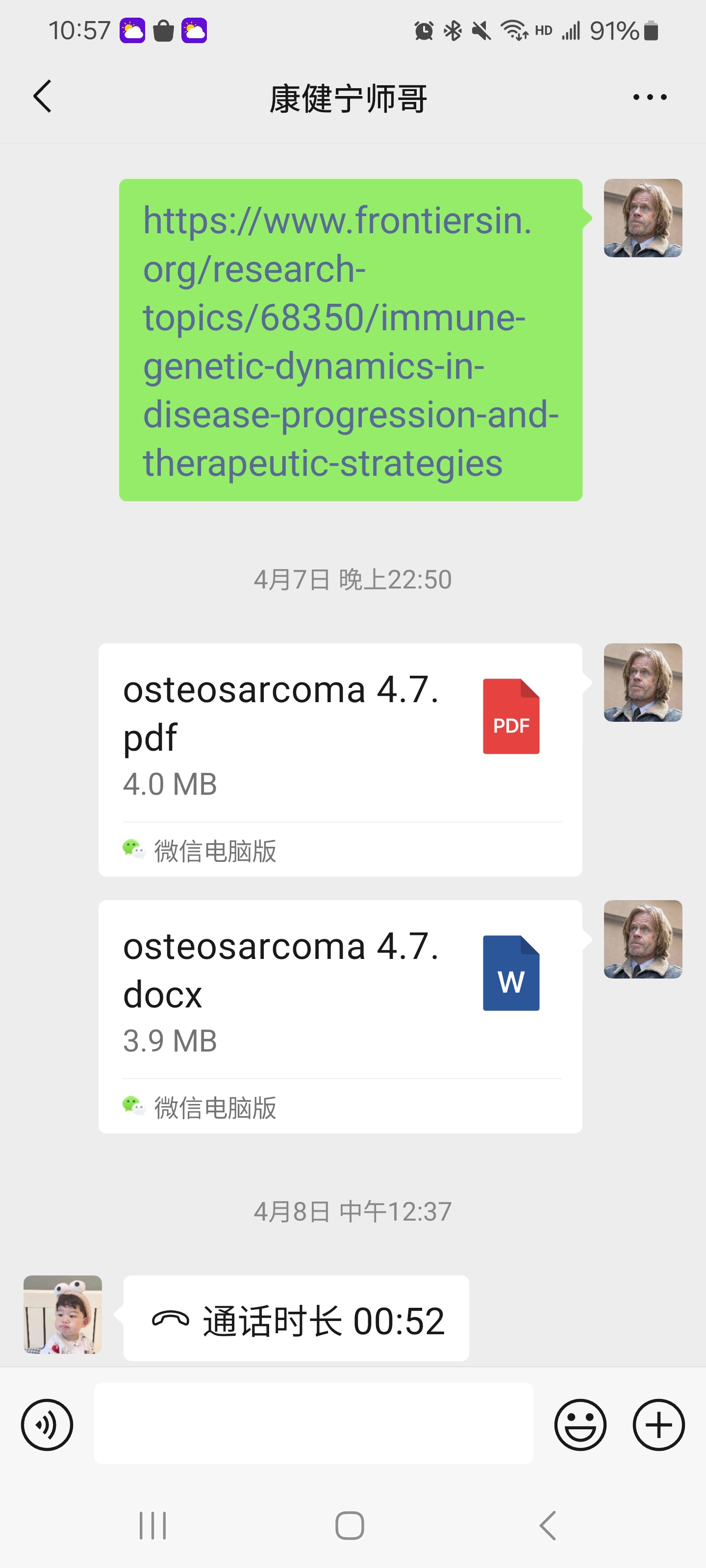

Supplement: Supplementary file 6 [file DataSheet1.zip › Authors and contributors/KJN (2).jpg]

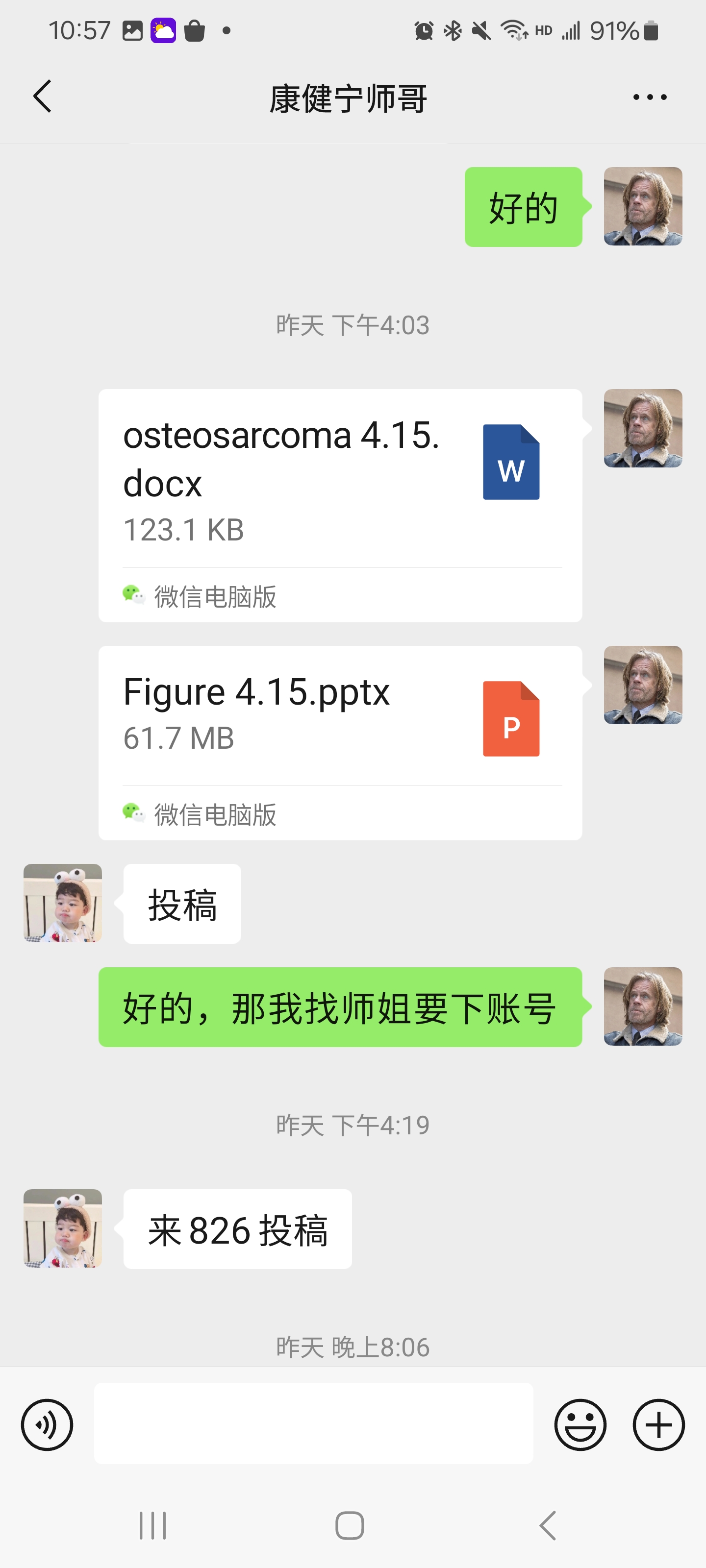

Supplement: Supplementary file 6 [file DataSheet1.zip › Authors and contributors/KJN.jpg]

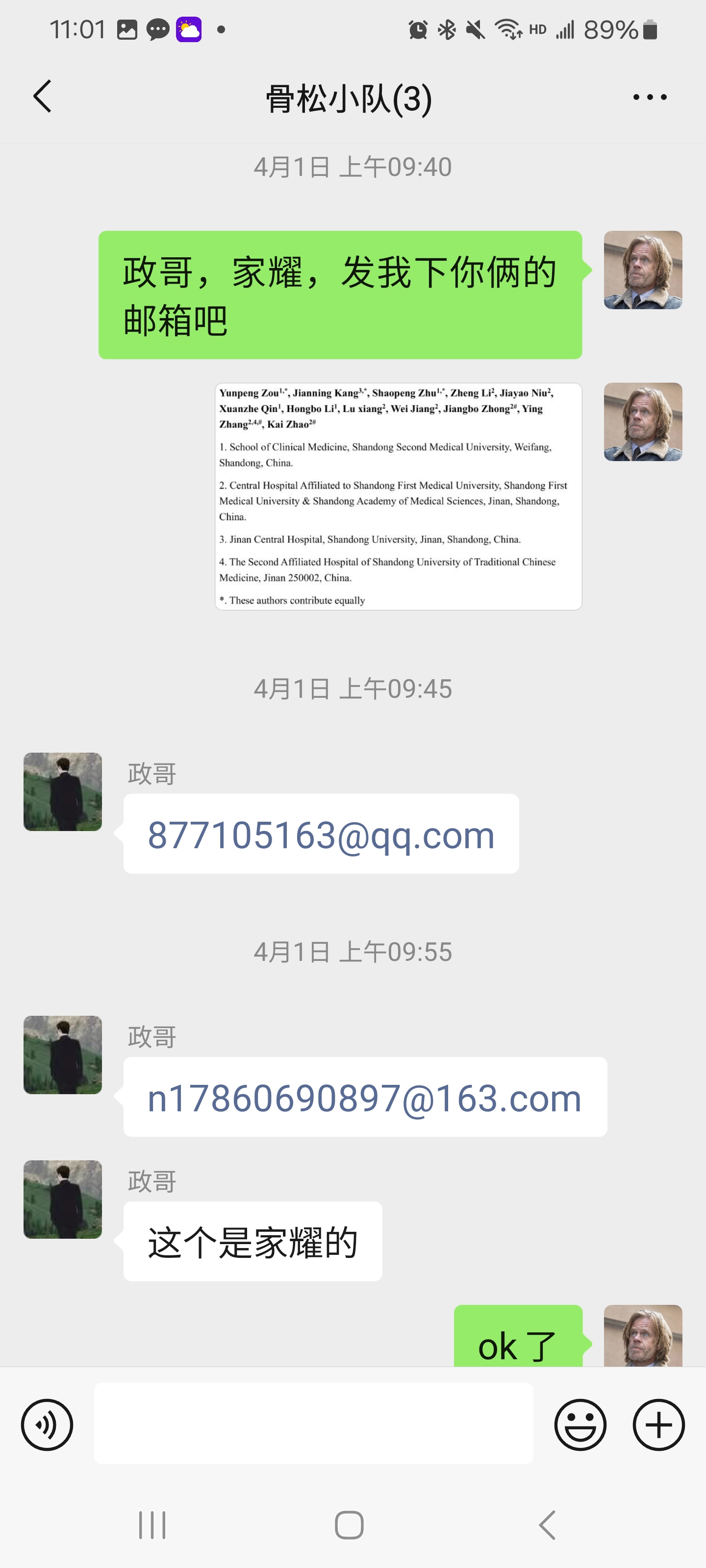

Supplement: Supplementary file 6 [file DataSheet1.zip › Authors and contributors/LZ&NJY.jpg]

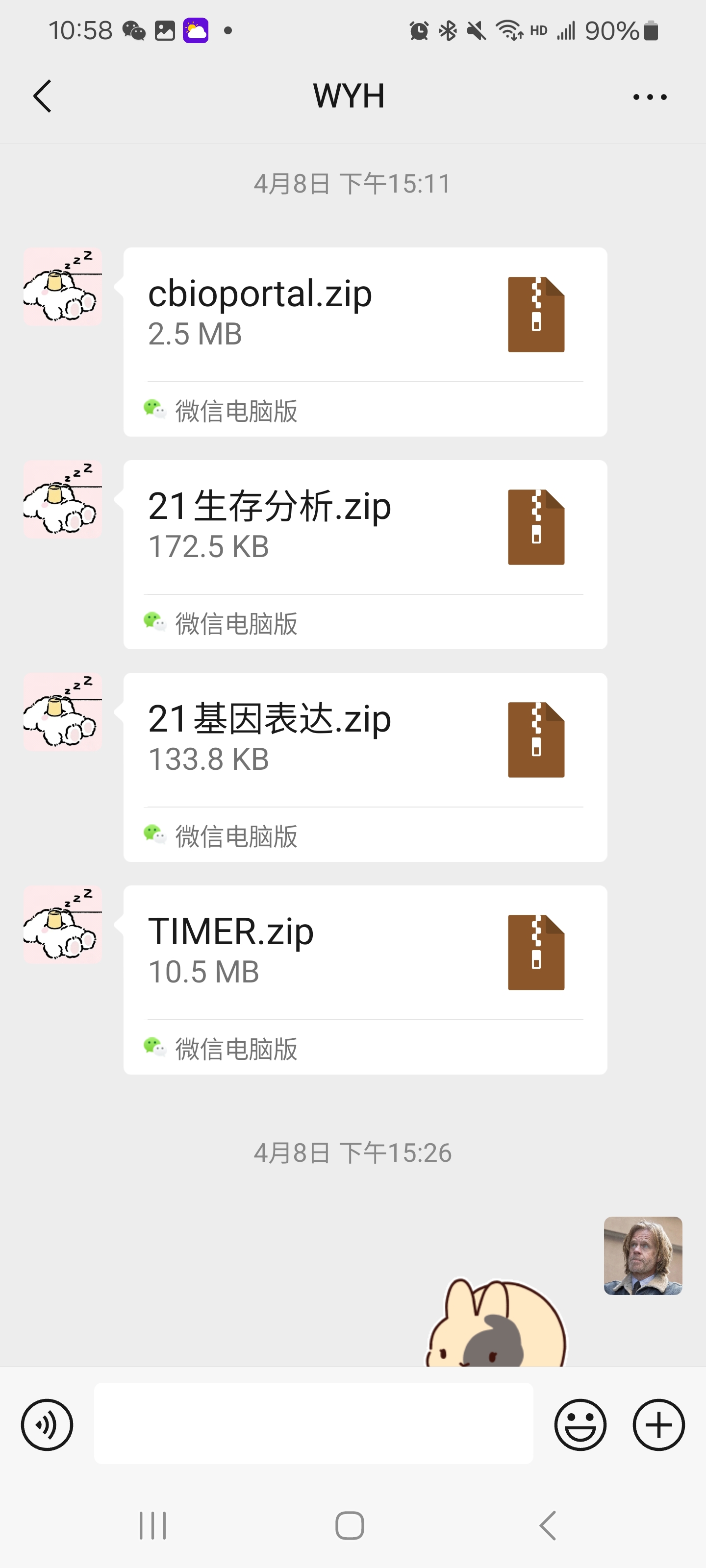

Supplement: Supplementary file 6 [file DataSheet1.zip › Authors and contributors/RXC.jpg]

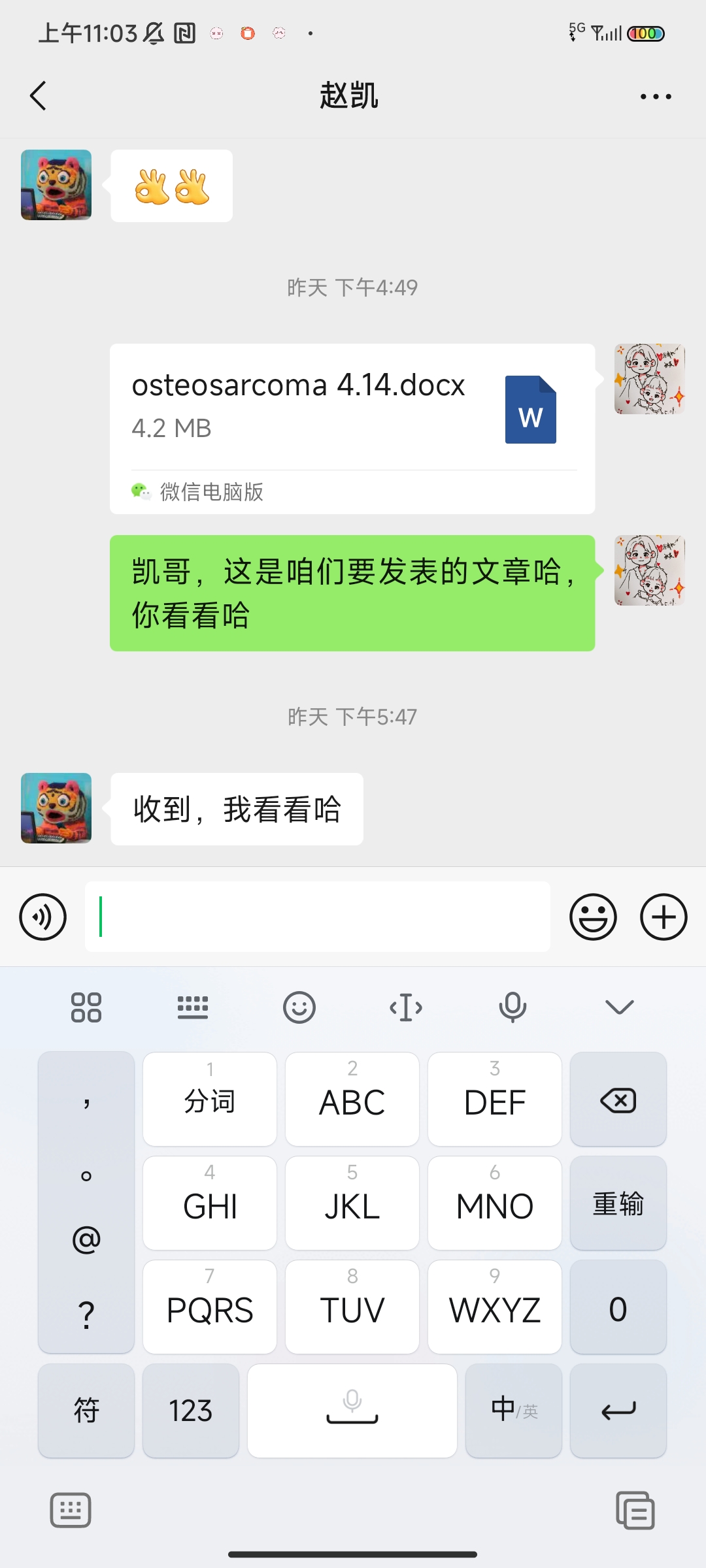

Supplement: Supplementary file 6 [file DataSheet1.zip › Authors and contributors/ZK.jpg]

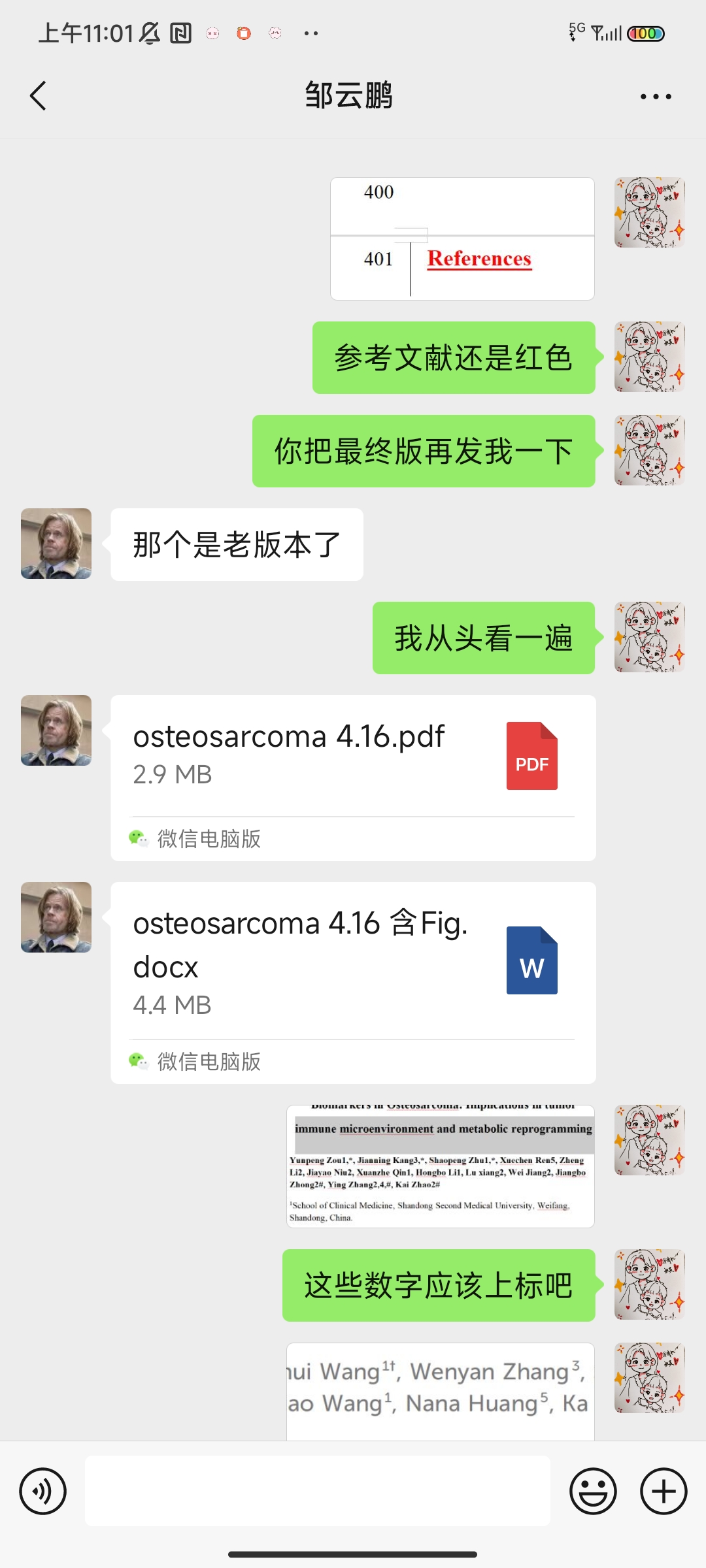

Supplement: Supplementary file 6 [file DataSheet1.zip › Authors and contributors/ZY (2).jpg]

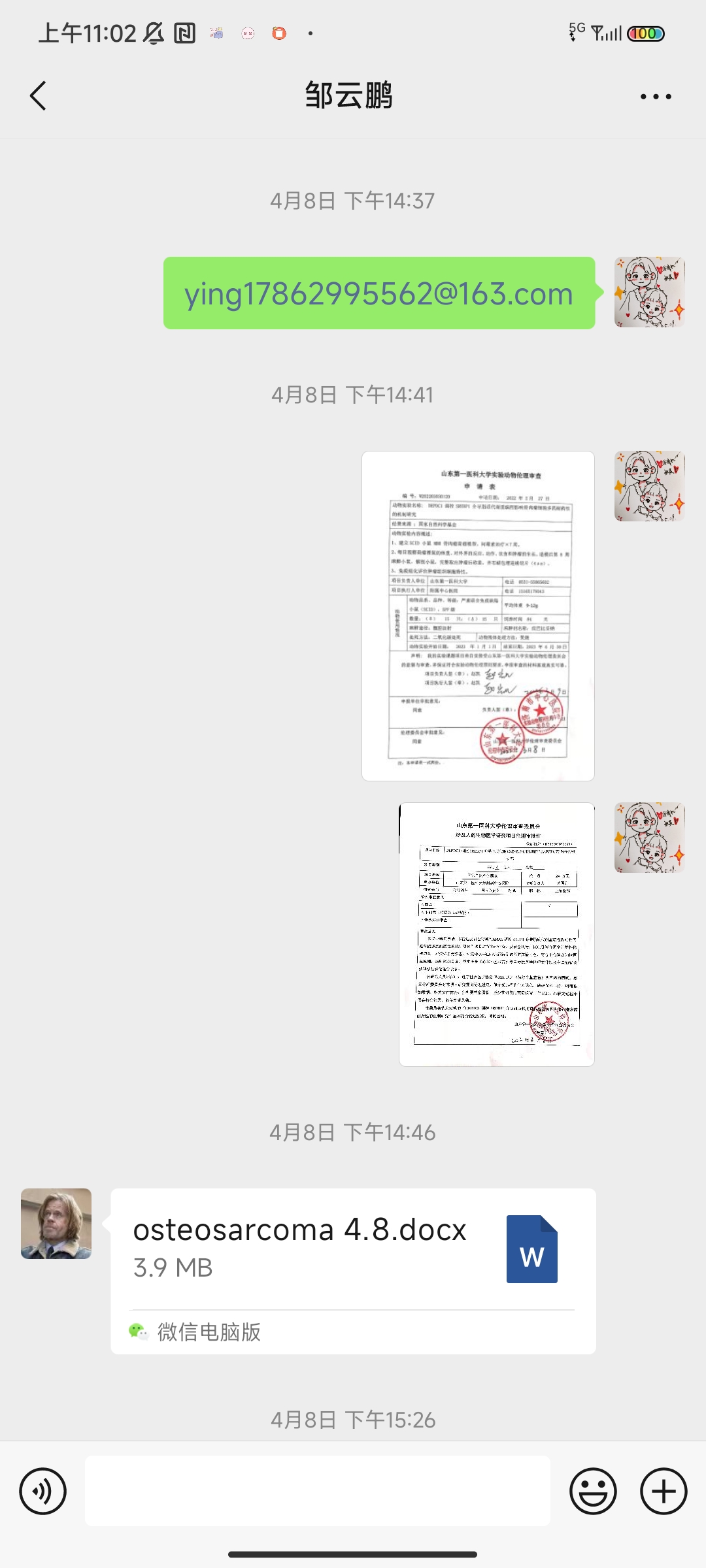

Supplement: Supplementary file 6 [file DataSheet1.zip › Authors and contributors/ZY.jpg]

Full unedited blot for Figure 9

marker 26616

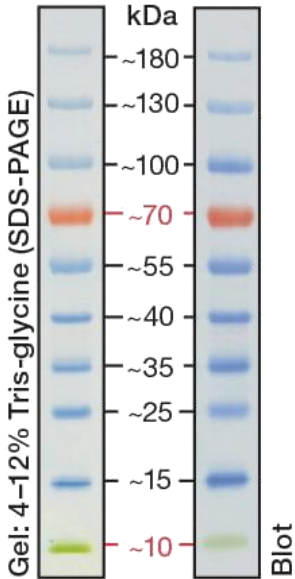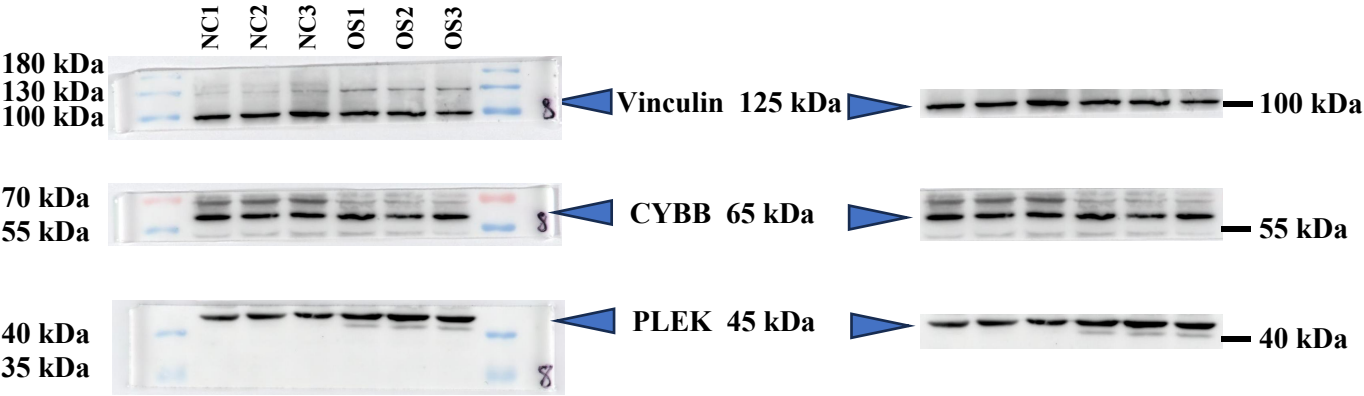

Full unedited blot for Figure 11

marker 26616

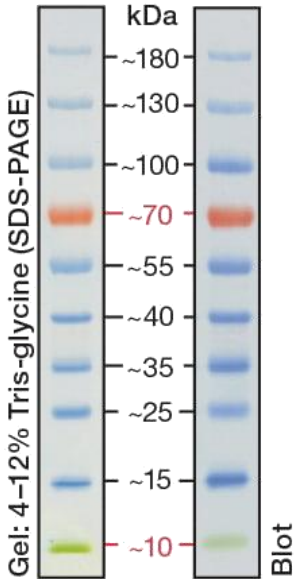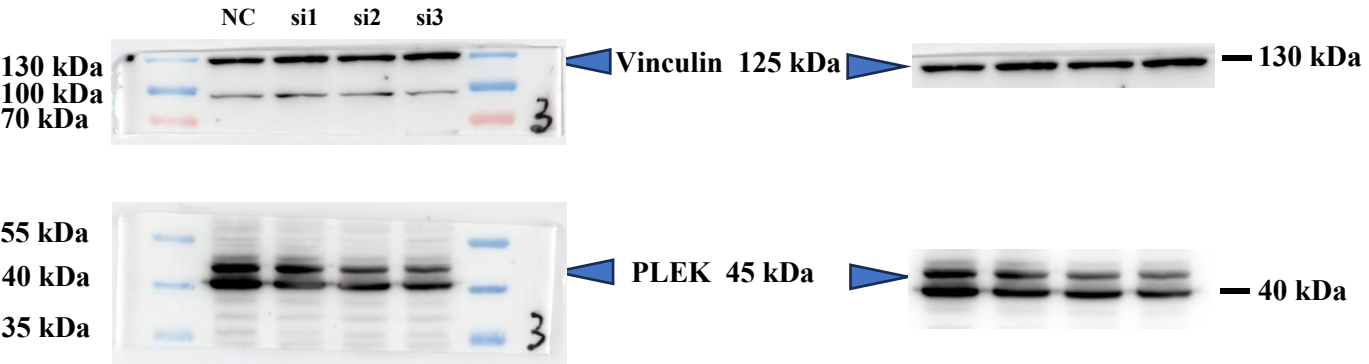

Supplement: Supplementary file 7 [file DataSheet2.pdf]
